# Global geological occurrence and character of the carcinogenic zeolite mineral, erionite: A review

**DOI:** 10.3389/fchem.2022.1066565

**Published:** 2022-11-18

**Authors:** Janki Prakash Patel, Martin S. Brook, Melanie Kah, Ayrton Hamilton

**Affiliations:** School of Environment, The University of Auckland, Auckland, New Zealand

**Keywords:** erionite, geology, review, formation, fiber morphology, distribution

## Abstract

As with the six regulated asbestos minerals (chrysotile, amosite, crocidolite, anthophyllite, tremolite, and actinolite), the zeolite mineral, erionite, can exhibit a fibrous morphology. When fibrous erionite is aerosolized and inhaled, it has been linked to cases of lung cancers, such as malignant mesothelioma. Importantly, fibrous erionite appears to be more carcinogenic than the six regulated asbestos minerals. The first health issues regarding erionite exposure were reported in Cappadocia (Turkey), and more recently, occupational exposure issues have emerged in the United States. Erionite is now classified as a Group 1 carcinogen. Thus, identifying the geological occurrence of erionite is a prudent step in determining possible exposure pathways, but a global review of the geological occurrence of erionite is currently lacking. Here, we provide a review of the >100 global locations where erionite has been reported, including: 1) geological setting of host rocks; 2) paragenetic sequence of erionite formation, including associated zeolite minerals; 3) fiber morphological properties and erionite mineral series (i.e., Ca, K, Na); and 4) a brief overview of the techniques that have been used to identify and characterize erionite. Accordingly, erionite has been found to commonly occur within two major rock types: felsic and mafic. Within felsic rocks (in particular, tuffaceous layers within lacustrine paleoenvironments), erionite is disseminated through the layer as a cementing matrix. In contrast, within mafic (i.e., basaltic) rocks, erionite is typically found within vesicles. Nevertheless, aside from detailed studies in Italy and the United States, there is a paucity of specific information on erionite geological provenance or fiber morphology. The latter issue is a significant drawback given its impact on erionite toxicity. Future erionite studies should aim to provide more detailed information, including variables such as rock type and lithological properties, quantitative geochemistry, and fiber morphology.

## 1 Introduction

Zeolites are volcanic in origin and are formed by the action of alkaline water or seawater on volcanic glass in sediments and clays. Of the more than 40 known zeolites, clinoptilolite is the most abundant in nature, followed by analcime, chabazite, heulandite, natrolite, phillipsite, mordenite, stilbite, and erionite (Reid et al., 2021). Zeolites have a very large internal surface area resulting from the particular configuration of their crystalline lattice. They can lose or gain water molecules and exchange cations without significant changes to their crystalline structure and have a catalytic activity (Dumortier et al., 2001). Erionite was first described by Eakle (1898) in its woolly form in Durkee, Oregon (United States). Erionite crystals can occur individually or as “radiating clusters,” “bundles,” or a “woolly mass” (Dogan and Dogan, 2008; Van Gosen et al., 2013). Occasionally erionite is found intergrowing with levyne, where it appears as short fibers growing in-between plates of levyne, and with offretite, where it forms stacking faults (Wise and Tschernich, 1976; Schlenker et al., 1977; Cametti and Churakov, 2020).

The morphology of erionite is the primary reason the mineral is toxic, and it is now known to exhibit three different compositions: calcium (erionite-Ca), sodium (erionite-Na), or potassium (erionite-K), as determined by the predominant element (Coombs et al., 1997; Dogan and Dogan, 2008). When aerosolized and inhaled, erionite fibers have been associated with health effects similar to those typically seen with exposure to asbestos, including malignant mesothelioma (Beaucham et al., 2018). In particular, the inhalation and respiration of erionite fibers were unequivocally linked to the malignant mesothelioma (MM) epidemic in the Cappadocia region of Turkey in the 1970s (Bariş et al., 1979, 1978; Artvinli and Bariş, 1979; Mumpton, 1979). The erionite was identified within the local soft, powdery surface rocks and led to the deaths of >50% in one village, Karain (Carbone et al., 2007). MM is a disease typically associated with environmental and occupational exposure to asbestos fibers (Hillerdal, 1983; Bianchi and Bianchi, 2007; Lacourt et al., 2014; Attanoos et al., 2018). However, *in vivo* studies conducted following the MM epidemic in Turkey suggested that erionite may be even more carcinogenic than crocidolite and chrysotile asbestos (Wagner et al., 1985; Coffin et al., 1992). Indeed, Coffin et al. (1992) proposed that erionite might be 500–800 times more tumorigenic than chrysotile asbestos, while Wagner et al. (1985) reported that 100% of rats inoculated with erionite died from MM. Subsequently, erionite has been recognized as a Group 1 Carcinogen by the International Agency for Research on Cancer (IARC, 2012, 1987).

**FIGURE 1:**
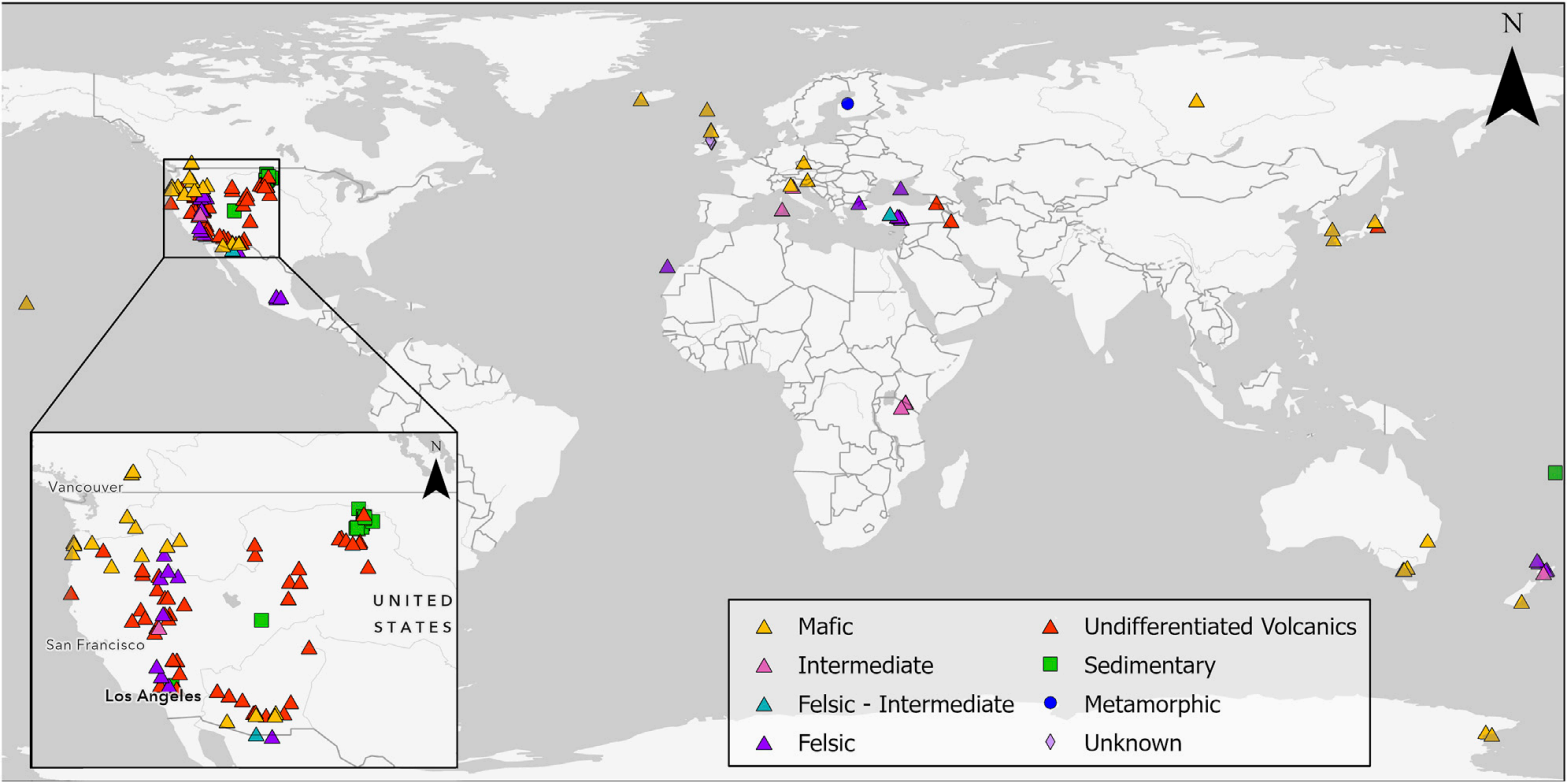
Global geological occurrence of erionite, with western North America, a particular focus of studies, shown in the inset map; sites are coded by geology. Details on the studies related to each location can be found in Supplementary Material S1.

**FIGURE 2:**
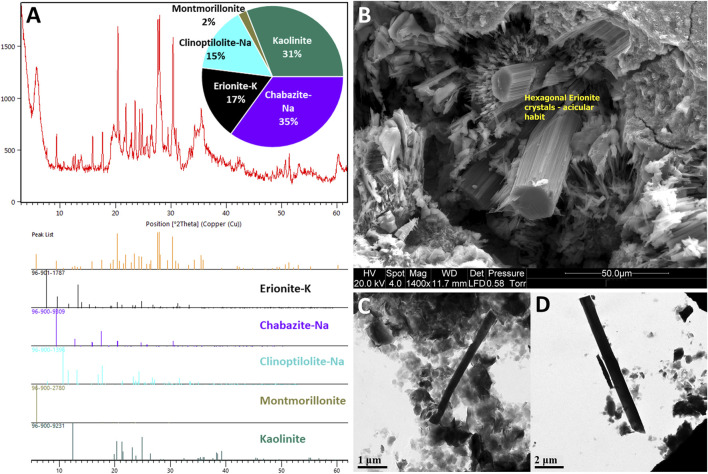
Example erionite-K from the Waitemata Group volcaniclastic sediments, Auckland, New Zealand (modified from Patel and Brook, 2021): **(A)** X-ray diffraction (XRD) plots showing erionite and other zeolites, including chabazite and clinoptilolite; **(B)** Scanning Electron Microscopy (SEM) image of example erionite fibers (yellow boxes), the fibers have formed hexagonal bundles with an acicular habit and formed within the pore spaces of siltstone undergoing weathering; **(C,D)** cryogenic Transmission Electron Microscopy (TEM) of example fibers to determine morphology.

Presently, cases of MM related to erionite exposure are restricted to Turkey (Bariş et al., 1979, 1978) and Mexico (Ortega-Guerrero et al., 2015), but due to its carcinogenic potential, there are concerns regarding the occupational and environmental exposure to erionite in other countries such as New Zealand (Patel and Brook, 2021), United States (Carbone et al., 2011; Beaucham et al., 2018), Iran (Ilgren et al., 2015) and Italy (Giordani et al., 2016). Indeed, erionite has been identified in various geological formations globally (Figure 1), and due to health concerns, significant research has been undertaken in the United States to identify the geological occurrences of erionite (e.g., Sheppard, 1996; Van Gosen et al., 2013) and in Italy (e.g., Giordani et al., 2017, 2016), although the latter study only focused on one region of Italy (the Lessini Mountains). Despite Giordani et al. (2017) listing some global erionite occurrences, a comprehensive review and synthesis of literature about the worldwide geologic occurrences of erionite is lacking. Indeed, the erionite literature mainly focuses on mineral chemistry (e.g., Gualtieri et al., 1998; Passaglia et al., 1998; Dogan and Dogan, 2008) and erionite toxicology (e.g., Wagner et al., 1985; Coffin et al., 1992; Dogan et al., 2006). For a mineral as toxic as erionite, delineation of the likely geological occurrence and geographic distribution can help inform exposure, while characterization of the mineral properties (habit, morphology) can be used to determine toxicity (WHO, 1986; Dogan et al., 2008; Beaucham et al., 2018). Both exposure and toxicity are essential for future risk assessments of erionite (Carbone et al., 2011; Giordani et al., 2017). Some examples of erionite from the Waitemata Group volcaniclastic sediments in Auckland, New Zealand, are shown in Figure 2, using a range of analytical approaches (discussed below).

Thus, given the interest in erionite relating to public and occupational health over the last 4 decades and the concomitant growth in journal publications, a review of the geological occurrence of erionite, including details on geological formation, rock type, and age, along with accompanying zeolite minerals, is pertinent and timely. The present study aims to review the global geological occurrence of erionite, including: 1) determining the principal rock types and environments in which erionite forms, 2) characterizing the properties of the reported erionite species, and 3) identifying the other zeolite minerals that erionite is commonly found alongside. To this end, the present study identified reports of erionite in 139 locations spanning 26 countries globally (Figure 1). Key aspects of the erionite reports are summarized in the accompanying figures and the Supplementary Material (Supplementary Table S1). The vast majority of published studies to date regarding erionite are not focused on geological aspects of the mineral occurrence, so the information in Supplementary Table S1 is somewhat fragmentary. Indeed, for locations where erionite was reported, only 95 out of the 139 studies described the basic morphology (e.g., acicular, woolly, or fibrous), while only 37 studies reported fiber size. The latter is critically important for any risk assessment (WHO, 1997).

## 2 The erionite mineral series

Erionite is a hexagonal-shaped tectosilicate belonging to the ABC-6 family of zeolites and consists of (Si, Al)O_4_ framework tetrahedra linked together to form single six rings and double six-rings, which create fibrous morphologies (Gottardi and Galli, 1985; Alberti et al., 1997; Gualtieri et al., 1998; Cametti et al., 2013; Giordani et al., 2016; Kshirsagar et al., 2021). Erionite consists of three types of cages: 1) an empty six-membered double ring, 2) a cancrinite cage preferred by K, and 3) an erionite cage with dispersed Ca, Na, and Mg cations (Gottardi and Galli, 1985; Gualtieri et al., 1998; Armbruster and Gunter, 2001; Ballirano et al., 2009; Giordani et al., 2016; Kshirsagar et al., 2021). In the early literature (e.g., Eberly, 1964; Sheppard and Gude, 1969; Gude and Sheppard, 1981), erionite was described as a single mineral, but subsequently, erionite was redefined as a series of minerals belonging to the erionite group (Coombs et al., 1997; Dogan and Dogan, 2008). The minerals within the erionite series are erionite-Ca (calcium), erionite-K (potassium), and erionite-Na (sodium), which are named based on the most abundant extra-framework cation within the mineral (Coombs et al., 1997; Dogan and Dogan, 2008; Dogan et al., 2008; Beaucham et al., 2018). Prior to 1997, the term erionite was used without a modifier, and even post-1997, many publications mention erionite without indicating which mineral within the erionite series is being referred to (e.g., Campbell et al., 2001; Ivanova et al., 2001; Anthony et al., 2003; Rodgers et al., 2004; Ilgren et al., 2015; Bernhart Owen et al., 2019; Kshirsagar et al., 2021). Therefore, unless referring to specific occurrences of erionite where chemistry is known, the term “erionite” will be used within this review paper without a modifier.

The general chemical formula for the erionite series, as defined by Coombs et al. (1997), is:

$$K_{2}{\left(Na,Ca_{0.5}\right)}_{8}[Al_{10}Si_{26}O_{72}].30H_{2}O$$

While Coombs et al. (1997) defined erionite into three minerals based on type localities for each mineral, Dogan and Dogan (2008) proposed a new general chemical formula for each erionite mineral based on the mean elemental values of erionite found in various areas. The formulae are as follows (Dogan and Dogan, 2008):

$$Erionite-Ca:\left({Ca}_{3.56}^{2+}K_{1.95}^{+}{Na}_{0.27}^{+}{Mg}_{0.30}^{2+}\right)\left({Si}_{25.78}{Al}_{10.28}{Fe}_{0.01}^{3+}\right)O_{72}$$

$$Erionite-Na:\left({{Na}_{4.00}^{+}K_{2.40}^{+}Ca}_{1.13}^{2+}{Mg}_{0.24}^{2+}\right)\left({Si}_{26.69}{Al}_{9.11}{Fe}_{0.22}^{3+}\right)O_{72}$$

$$Erionite-K:\left(K_{2.80}^{+}{{Na}_{1.66}^{+}Ca}_{1.03}^{2+}{Mg}_{0.51}^{2+}\right)\left({Si}_{28.21}{Al}_{7.39}{Fe}_{0.41}^{3+}\right)O_{72}$$

Furthermore, Dogan and Dogan (2008) have also stated that for a zeolite mineral to be classified as erionite, it must pass both the balance error and Mg content tests. The balance error formula is:

$$E=\frac{\left[\left(Al+{Fe}^{3+}\right)-\left(Na+K\right)+2\left(Ca+Mg\right)\right]}{\left[\left(Na+K\right)+2\left(Ca+Mg\right)\right]}\times 100$$

From this formula, a mineral can only be classified as erionite if the balance error (E%) is less than or equal to 10% (Passaglia, 1970; Dogan, 2003; Dogan and Dogan, 2008). The Mg^2+^ content must also not exceed 0.80 atoms per cell, and if it does, then the mineral will also not be characterized as erionite (Gualtieri et al., 1998; Dogan and Dogan, 2008). In addition to the E balance error and Mg content tests outlined by Dogan and Dogan (2008), Cametti et al. (2013) have also drawn attention to extra framework cations, including K. Cametti et al. (2013) suggest that if the K atom, which lies at the K1 site located at the center of the cancrinite cage has a value of less than 2K apfu, then this indicates a partly vacant K1 site, or more plausibly, an incorrect analysis. Thus, the content of K could also be used, in addition to the E balance error and Mg content, in order to assess the quality of analytical results. Chemically classifying erionite is prudent as the morphology of erionite is similar to other fibrous zeolites, such as offretite. The corollary is that morphology alone should not be used to determine if a zeolite is actually erionite and that the chemistry is fundamentally important (Passaglia et al., 1998; Dogan and Dogan, 2008; Cametti et al., 2013).

**FIGURE 3:**
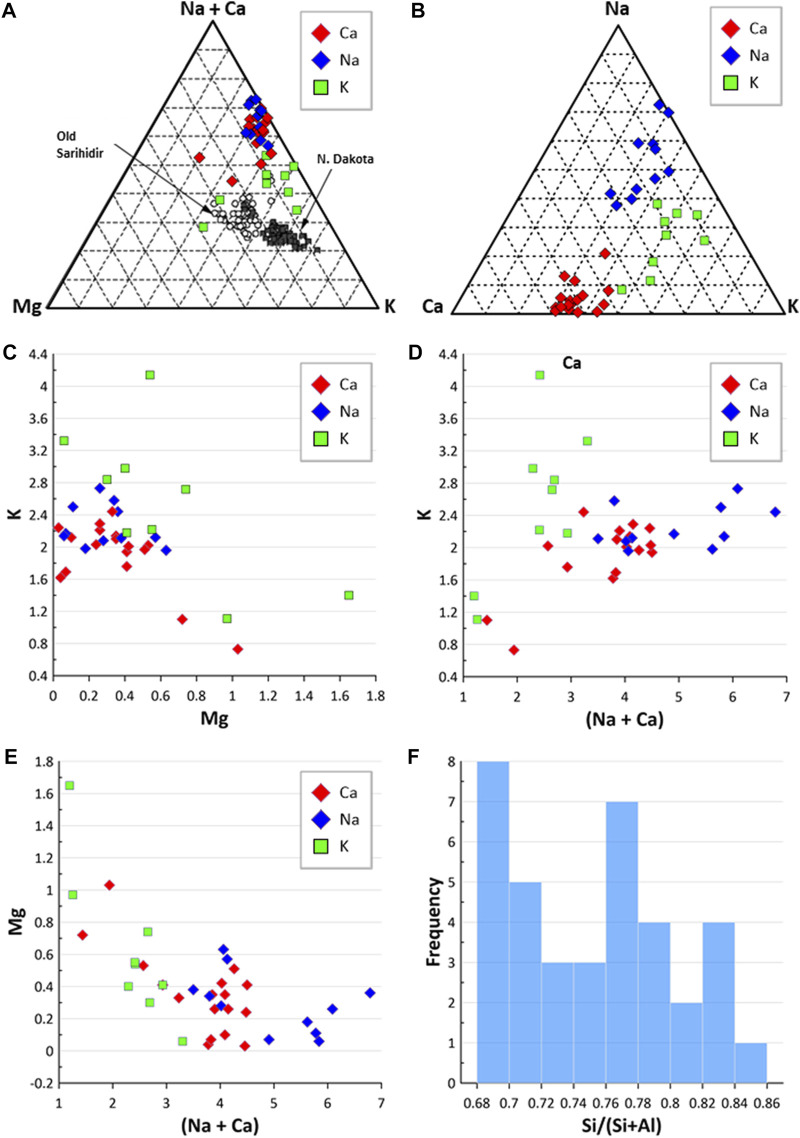
Erionite chemistry. **(A)** Ternary diagram comparing erionite global geological occurrence to erionite from N. Dakota and Old Sarihidir (following Carbone et al., 2011); **(B)** ternary diagram of Na-C-K; **(C)** Mg and K composition of different erionite species; **(D)** comparison of (Na + Ca) values against K; **(E)** comparison of (Na + Ca) against Mg; **(F)** frequency of Si/(Si + Al) ratios.

Of the 139 locations shown in Figure 1 (and reported in Supplementary Table S1) where erionite has been reported, only 38 reported the chemical composition of the erionite minerals in any detail. This erionite chemistry data is summarized in Figure 3, using the three end-members (K, Ca, Na) and Mg. The cations Sr and Ba were omitted as they are minor components of erionite (e.g., Passaglia et al., 1998; Dogan and Dogan, 2008; Carbone et al., 2011). Figure 3 displays the chemistry of erionite from the various published locations around the world, as shown in Figure 1, with the majority of the chemical data coming from Dogan and Dogan (2008). Figure 3A is a conventional K-Mg-(Ca+Na) ternary diagram, following Carbone et al. (2011), who attempted to use such a ternary diagram to infer that there was only a small difference between the chemical characteristics of Turkish and North Dakota (ND) erionite. In Figure 3A, differences seem apparent between the composition of Ca, Na, and K erionite reported globally, with erionite-K dispersed on the ternary plot away from Ca and Na erionite, which are more clustered. For comparison, the global dataset is shown in a somewhat simpler K-Ca-Na ternary plot in Figure 3B, and the Ca, N, and K erionite differences are more equivocal than in Figure 3A. Carbone et al. (2011, p. 13619), compared the chemistry of erionite from ND and Old Sarihidir and concluded that “in summary, our data show that the….chemical characteristics of Turkish and ND erionite are very similar.” However, while the ND and old Sarihidir datasets do overlap in Figure 3A, there is considerable dispersion, which is also suggested by their accompanying bar chart (their Figure 2). Carbone et al. (2011, p. 13621) disclosed that their “analyses have been adjusted for calculated Na loss and thus appear closer to the vertex relative to K” without detailing the nature of their adjustments. Carbone et al. (2011, p.13261) then go on to state that “there is no *a priori* reason….to suggest that this small difference [in chemistry] will affect the carcinogenicity of the erionite.” This is despite their ternary plot and bar chart (their Figure 2), implying that the difference in Old Sarihidir and ND erionite chemistry *is* not minor. This (apparently) overlooked difference in erionite chemistry is important because Carbone et al. (2011) then undertook biological activity (foci development *via* cell coculture testing) testing of the ND and Old Sarihidir erionite, applying the Student’s t-test (e.g., Bland, 1995). From the coculture testing, Carbone et al. (2011, p. 13621) then concluded, “our data show that ND erionite was more potent than Cappadocian erionite in inducing foci formation.” Indeed, the Cappadocian erionite showed 3× the number of foci after 3 months than the ND erionite (Carbone et al., 2011, p. 13622). The corollary is that the differences in chemistry may be an important influence on carcinogenicity, yet appears to have been discounted by the authors. Moreover, erionite chemistry datasets reported from global erionite occurrences (see Supplementary Table S1) are also superimposed on Figure 3A, and appear to reveal that the two datasets reported by Carbone et al. (2011) are enriched in K and Mg relative to global data. Thus, it would appear that Carbone et al. (2011) may also have (unwittingly) introduced a statistical bias into the plotting of their data, rendering the applicability of their use of a ternary diagram to infer similarities between the ND and Old Sarihidir datasets, questionable. Such issues of using ternary diagrams have long been debated in the geochemistry literature. For example, Butler’s (1979) analysis of 114 igneous rocks in Texas showed that re-casting variables into percentages within a ternary diagram format dramatically changed the statistical properties of the data in that the variable with the smallest variance in the initial set-up had the largest variance in the ternary data set-up. Indeed, the formation of ternary diagram percentages induces closure into the data, so that an unknown amount of the depicted variability is actually an artifact of the closure (Butler, 1979). Thus, bivariate scatterplots (e.g., Figures 3C–E) or simple frequency histograms (Figure 3F) alongside discriminant functions may be more appropriate approaches to displaying erionite chemistry and inferring genetic trends of different erionite minerals. Indeed, globally the Si content varied across many locations but commonly was in the range of 0.68–0.72 and 0.76–0.80 (Figure 3F). These are very similar value ranges to the Si content for North Dakota and Old Sarihidir, as reported by Carbone et al. (2011).

## 3 Geology

### 3.1 Global occurrence

Erionite has been found worldwide in many different countries, as shown in Figure 1, yet often in publications, much of the key geological data useful in characterizing erionite (e.g., rock units, paleoenvironment, apparent mode of formation) is missing or incomplete. Notwithstanding these limitations, Italy, Turkey, and the United States are three countries where in-depth analyses into the geological occurrence and characterization of erionite has occurred (Artvinli and Bariş, 1979; Sheppard, 1996; Giordani et al., 2017). In the United States, Sheppard’s (1996) widely-cited work focused only on one geological environment (sedimentary rocks). Subsequently, a more detailed USA-focused geological review of erionite was published by Van Gosen et al. (2013) from the standpoint of an emerging national public health concern for respiratory disease. Elsewhere, global data on erionite is less abundant, but locations, where erionite has been reported include Antarctica (Vezzalini et al., 1994), Australia (England and Ostwald, 1979; Birch, 1987), Fiji (Ram et al., 2019), Finland (Lehtinen, 1976), Georgia (Batiashvili and Gvakharia, 1968), Crimea (Suprychev and Prokhorov, 1986), Scotland (Macpherson and Livingstone, 1982), Northern Ireland (Passaglia et al., 1998), Japan (Harada et al., 1967; Kawahara, 1967; Shimazu and Kawakami, 1967), Kenya (Surdam and Eugster, 1976; Bernhart Owen et al., 2019), Austria (Zirkl et al., 1962; Waltinger and Zirkl, 1974), and New Zealand (Sameshima, 1978; Irwin, 2016; Patel and Brook, 2021). Erionite has been found within rocks used for road aggregates in the United States (Van Gosen et al., 2013), and in the rock used to construct houses in Turkey (Carbone et al., 2007). Hence understanding the geological occurrence, formation processes, and geographic distribution of erionite-bearing rock is important.

### 3.2 Geological settings of erionite

**FIGURE 4:**
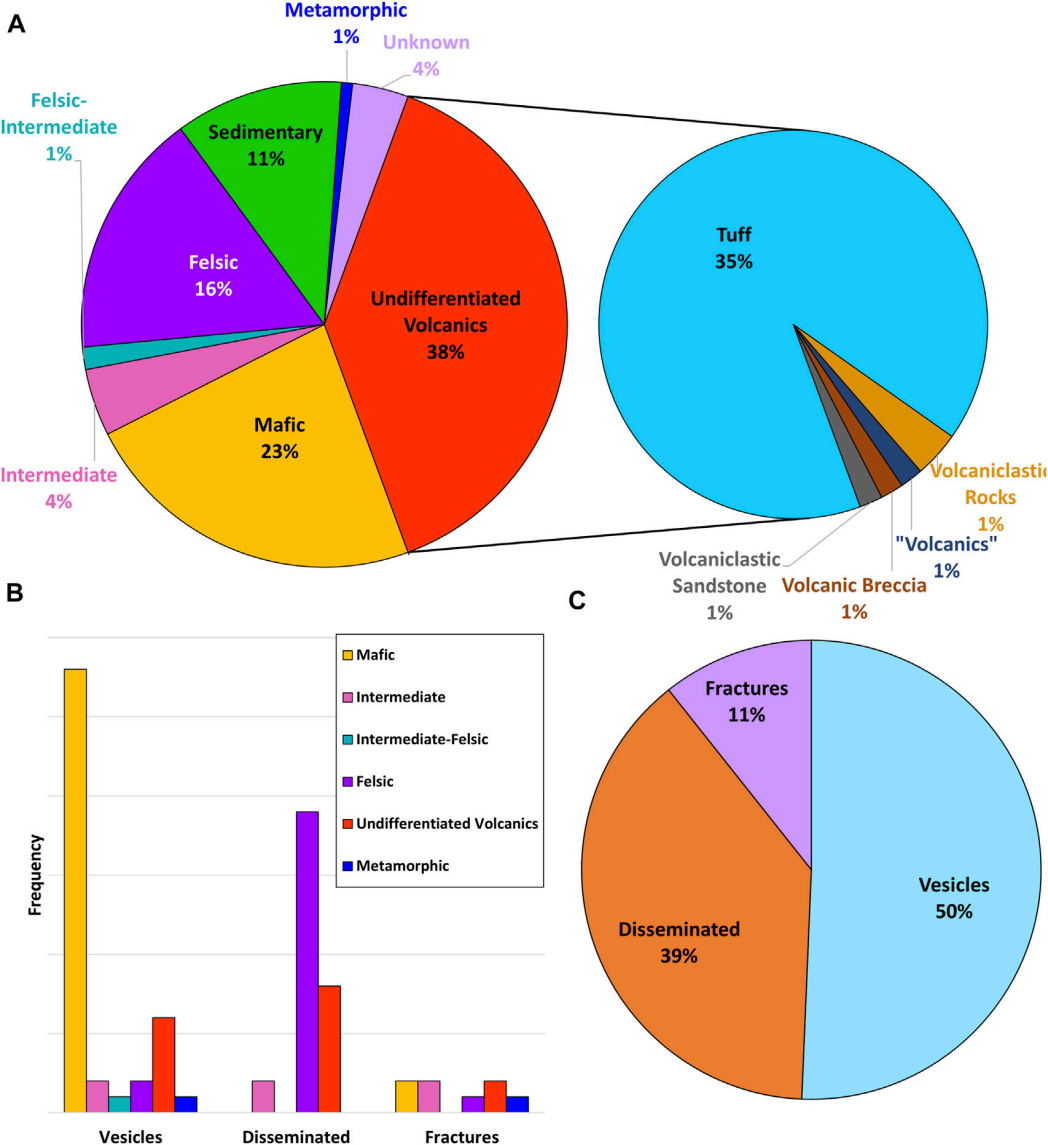
Properties of erionite bearing rock. **(A)** Rock types erionite has been found in (often tuff type is not differentiated within the literature); **(B)** where erionite is found within the rocks; **(C)** overall proportions of erionite reported from vesicles, fractures, and disseminated within the rock mass (note: vesicles refer to vesicles, vugs, and amygdules; disseminated refers to matrix; fractures includes both veins and fractures).

As with other zeolites, erionite is usually identified within volcanic and volcanically-derived rocks (Figures 1, 4A), where the minerals typically form *via* diagenesis or hydrothermal alteration (Mumpton, 1979; Coombs et al., 1997). Nevertheless, erionite has also been identified in sedimentary and metamorphic rocks (Lehtinen, 1976; Sheppard, 1996; Schmieder and Jourdan, 2013). The characteristic host rocks that erionite typically occurs in are basalt and tuffs, but for this review, the volcanic rocks have been classified based on their wt% SiO_2_ composition. The classification *via* SiO_2_ is because when describing certain rock types, such as tuff, the definitions can vary according to the author. For example, the erionite in Cappadocia (Turkey) was from, *per se loquendo*, rhyolitic pyroclastic deposits, yet these same deposits have been referred to as both welded tuff (e.g., Wagner et al., 1985; Topal and Doyuran, 1998) and ignimbrite (Temel and Gündoğdu, 1996). Moreover, the use of the terms “felsic” (silica-rich) and “mafic” (Mg and Fe-rich) are consequently more useful from a geological standpoint (e.g., Marshak, 2019). Unfortunately, however, many erionite-related publications are not focused on geology, but rather on health and toxicity, so even rudimentary geological information is often absent. Thus, as the majority of publications reviewed did not specify host rock composition, these rocks were classified as “undifferentiated volcanics” (Figure 1), with the majority of these undifferentiated volcanics assumed to be “tuff” (Figure 4A). Nevertheless, the host rock type is important, as it can indicate possible erionite fiber size. Indeed, larger well-formed erionite crystals are frequently found within the cavities and veins of volcanic rocks, while fine-grained crystals are more often homogenously distributed within volcanoclastic or sedimentary rocks (Rakovan, 2004; Marantos et al., 2012). Indeed, most larger fibers (>1 mm) are reported from mafic rocks, typically crystallizing within vesicles (e.g., Kamb and Oke, 1960; Tschernich and Wise, 1982; Passaglia and Tagliavini, 1995). Fiber size is discussed in more detail in Section 4.1 below.

**FIGURE 5:**
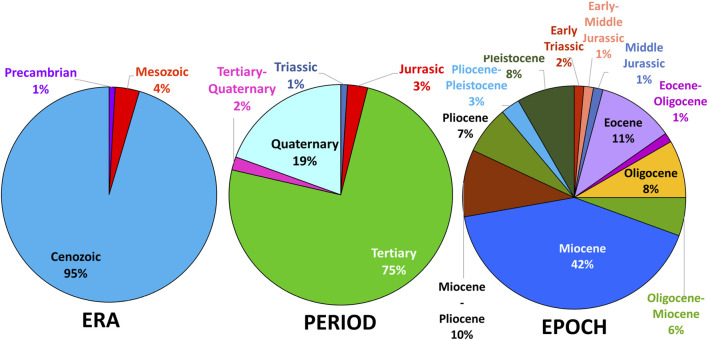
Age of erionite host rocks, classified by geological era, geological period, and geological epoch. The majority of rocks that erionite has crystallized in are younger rocks formed in the Cenozoic Era, primarily within the Miocene (i.e., 23–5.3 Ma).

From the available literature, within mafic rocks, erionite tends to form within vesicles (e.g., Hey, 1959; Wise and Tschernich, 1976; England and Ostwald, 1979; Rychly et al., 1982; Bargar and Keith, 1984; Noh and Kim, 1986; Birch, 1987; Vezzalini et al., 1994). In contrast, for felsic rocks, most erionite was reported within the matrix of the rock (Figure 4B; e.g., Cochemé et al., 1996; Donoghue et al., 2008; Eyde and Irvin, 1979; Hay, 1964; Mumpton, 1979), but can also be found to form within vesicles (e.g., Barrows, 1980). Most reported erionite occurrences are in vesicles or disseminated within a sedimentary layer, and only very rarely was erionite reported in a vein (Figure 4C). According to Marantos et al. (2012), zeolites forming within sediments typically form the cementing matrix, crystallizing within pore spaces, and this can lead to a harder zeolitized horizon layer, relative to the underlying and overlying sedimentary layers (Davidson and Black, 1994), and therefore are more resistant to erosion. A further intriguing geological factor is the age of the host rock (Figure 5). Most erionite globally is reported from rock units formed during the Miocene epoch (23–5.3 Ma), but erionite has been reported from rocks across a range of geological timescales. However, although the timing of zeolitization must post-date the rock unit age, the exact timing or duration is unknown in most cases. For example, erionite is found within Pleistocene-age (<2.58 Ma) rocks in New Zealand (Rodgers et al., 2004), as well as Precambrian age rocks (1.90–1.87 Ga), such as at Lake Lappajärvi, Finland (Lehtinen, 1976). At Lake Lappajärvi, zeolitization probably occurred much more recently during the late Cretaceous, following the meteorite impact event at ∼77.9 Ma (Kenny et al., 2019).

### 3.3 Paleoenvironments and formation processes

Erionite (and zeolites more broadly) typically form from diagenesis or hydrothermal alteration, crystalizing from fluids present within the host rock (Giordani et al., 2017; Patel et al., 2022). When hydrothermal alteration causes zeolitization, the conditions include low pressure and temperatures (<110°C). Examples of such locations where this process has occurred include Cairns Bay, Australia (Birch, 1988) and Eastern Rhodopes, Bulgaria (Ivanova et al., 2001; Kirov et al., 2011). Another typical mechanism of zeolite formation is dissolution *via* diagenesis, which has occurred in a variety of locations globally, including Guanajuato, Mexico (Ortega-Guerrero and Carrasco-Núñez, 2014; Ortega-Guerrero et al., 2015), Reese River, United States (Gude and Sheppard, 1981) and Chojabaru, Japan (Shimazu and Mizota, 1972). Note that for many published studies, the erionite formation processes were not reported by the authors, which, again, limits the possibility of accurate geospatial mapping of potentially hazardous erionite-bearing units. Notwithstanding this, from the literature summarized in Supplementary Table S1, six characteristic geological settings can be recognized for erionite formation, outlined below.

(1) Hydrothermal alteration of silica-rich volcanic deposits—heated hydrothermal fluids alter the surrounding host rock and cause the precipitation of erionite and other minerals (Bargar and Beeson, 1981; Van Gosen et al., 2013). Primarily the fluids are heated from below, and typically the temperature to crystallize erionite is low at around <110°C and can be found within vesicles and fractures of volcanic rocks (Bargar and Beeson, 1981). Examples include sinters such as at Otamakokore, New Zealand (Rodgers et al., 2004), and geysers at Yellowstone, United States (Honda and Muffler, 1970; Bargar and Beeson, 1981). Typically, such host rocks are young and of Pleistocene age (∼2.58 Ma; Bargar and Beeson, 1981; Rodgers et al., 2004).
(2) Diagenesis within lacustrine paleoenvironments—volcanic ash settled into lakes of a primarily alkaline composition (Temel and Gündoğdu, 1996; Van Gosen et al., 2013; Karakaya et al., 2015). Diagenesis occurred within this environment, with zeolites crystalizing from the dissolution of volcanic glass in the ash beds (Cochemé et al., 1996; Giordani et al., 2017). Some zeolite crystallization occurred within a closed system, primarily from ash layers interbedded between mudstone and claystone, or from shallow burial (Gude and Sheppard, 1988; Sheppard, 1991). Examples of lacustrine paleoenvironments which led to the crystallization of erionite include Mud Hills, California, United States (Sheppard and Gude, 1969; Sheppard, 1996; Van Gosen et al., 2013), Cappadocia, Turkey (Temel and Gündoğdu, 1996; Giordani et al., 2017), and Agua Prieta, Mexico (Cochemé et al., 1996; García-Sosa and Solache Ríos, 1997).
(3) Diagenesis within mafic rocks—primarily within these locations, erionite formed as lining within vesicles and fractures in basalt (Bennett and Grose, 1978; Noh and Kim, 1986). Diagenesis occurred due to groundwater percolation through the host rock, causing the alteration and crystallization of zeolites (Bennett and Grose, 1978). Examples of locations are Yeongil, South Korea (Noh and Kim, 1986), Chojabaru, Japan (Shimazu and Mizota, 1972), and Beech Creek, United States (Sheppard et al., 1974; Bennett and Grose, 1978). These host rocks are from the Cenozoic Era (<66 Ma).
(4) Hydrothermal alteration of intermediate to mafic rocks—leads to zeolitization within cavities and veins (Vezzalini et al., 1994; Giordani et al., 2017). Within this type of geological setting, hydrothermal fluids have caused the precipitation of zeolites, with rock ages ranging from Jurassic (201–145 Ma) to Cenozoic (<66 Ma) Era (Birch, 1988; Vezzalini et al., 1994). Examples of locations include dolerites from Mount Adamson, Antarctica (Vezzalini et al., 1994), basalt from Cairns Bay, Australia (Birch, 1988), and basalt from Lessini Mounts, Italy (Mattioli et al., 2016; Giordani et al., 2017).

In addition, two further modes of formation are occasionally reported within the literature and include 5) diagenesis in a marine environment such as in Auckland, New Zealand (e.g., Davidson and Black, 1994), and 6) hydrothermal alteration *via* meteorite impact metamorphism (e.g., Lake Lappajärvi, Finland; Lehtinen, 1976; Schmieder and Jourdan, 2013).

Occasionally, there is discord within the literature about how erionite minerals (and associated zeolites) have formed. For example, two contrasting formation processes have been proposed for erionite formed within the Miocene Waitemata Group in Auckland, New Zealand (Sameshima, 1978; Davidson and Black, 1994). According to Sameshima (1978), zeolites formed within a bathyal submarine environment *via* hot spring activity, accompanied by hydrothermal alteration at a shallow burial depth. In contrast, Davidson and Black (1994) proposed that zeolites formed due to diagenesis within a closed hydrologic system, based on the premise that zeolitization was confined to very specific lithological layers. Thus, because zeolites appeared restricted to specific sedimentary layers, rather than being disseminated throughout the surrounding units, the hot spring theory by Sameshima (1978) was deemed to be incorrect.

A further example of conjecture within the literature concerns erionite found in southern Bulgaria, which Ivanova et al. (2001) proposed was formed from low-temperature hydrothermal solutions, heated by hot pyroclastic material (i.e., hydrothermal alteration). In contrast, Kirov et al. (2011) more recently proposed that zeolitization occurred from diagenesis within a closed system. Both hypotheses may be valid as Kirov et al. (2011) explained that temperatures may have risen due to later volcanism, causing hydrothermal fluids to alter host rocks.

### 3.4 Erionite paragenesis

**FIGURE 6:**
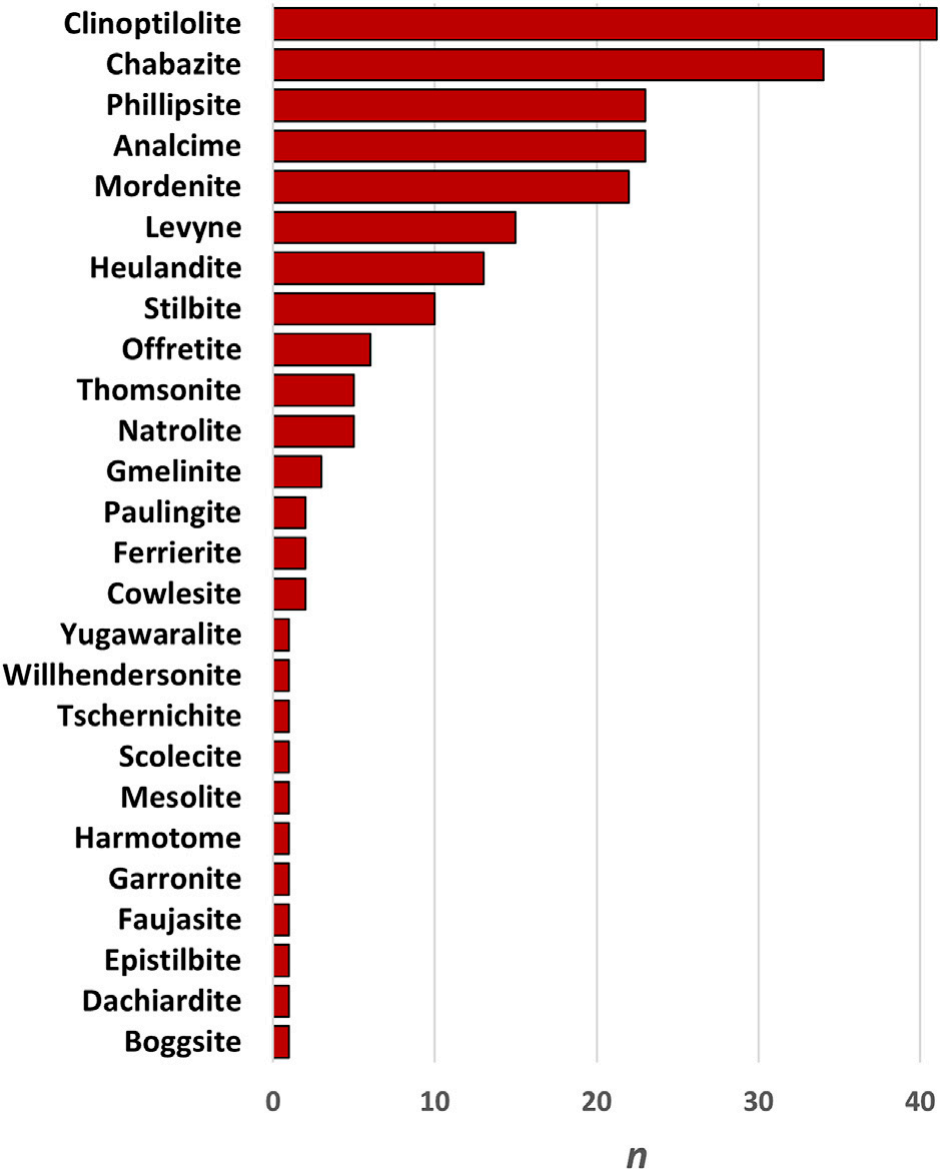
Frequency chart of other zeolites reported to occur alongside erionite, with clinoptilolite and chabazite being the most prevalent.

The paragenetic sequence of zeolite crystallization within a rock mass can provide insights into the environment that the zeolites formed in because the sequence of zeolite mineral formation is indicative of both the fluid chemistry and host rock chemistry present within the system (Hay, 1963; Surdam and Eugster, 1976; Davidson and Black, 1994; Kirov et al., 2011; Mattioli et al., 2016). The most common zeolites forming alongside erionite are clinoptilolite, chabazite, phillipsite, analcime, and mordenite (Figure 6). Observations from Surdam and Eugster (1976) at Lake Magadi, Kenya, found that erionite was formed in environments that are silica and sodium-rich, but which are low in calcium. Indeed, the most common mineral assemblages within the High Magadi beds are erionite + analcime ± quartz ± magadiite and erionite + analcime + chabazite + quartz. Therefore, the first zeolite to form is erionite, which forms straight from trachytic glass with the addition of H_2_O. Furthermore, analcime indicates a sodium-rich environment (Surdam and Eugster, 1976; Davidson and Black, 1994). However, as erionite-Ca is a mineral within the erionite series, the observations by Surdam and Eugster (1976) are specific for that area, given that to form a calcium end-member, calcium would need to be abundant within the host rock and/or fluid.

In contrast, Birsoy (2002) provided a broader definition for erionite occurrence, suggesting that erionite forms in alkaline environments. In particular, three locations specifically reported high pH (≥7) levels, including Kandovan, Iran (Ilgren et al., 2015), Tierra Blanca, Mexico (Ortega-Guerrero and Carrasco-Núñez, 2014), and Tuzgölü Basin, Turkey (Karakaya et al., 2015). Moreover, many other studies have simply referred to an alkaline environment, such as Ashio Tochigi, Japan (Matsubara et al., 1978), Olduvai Gorge, Tanzania (Hay, 1963; McHenry et al., 2020), and the studies from the United States in Durkee, Rome (Staples and Gard, 1959), Eastgate, Nevada (Papke, 1972; Sheppard, 1996), Kildeer Mountain, North Dakota (Goodman and Pierson, 2010; Saini-Eidukat and Triplett, 2014), and Reese River, Nevada (Deffeyes, 1959; Gude and Sheppard, 1981). Taken together, the reports indicate that erionite will likely form within an alkaline-rich environment. Such environments tend to be lacustrine, rather than marine, with alkaline lakes usually found in quiescent or recently extinct volcanic areas where neither water vapor nor acidic magmatic gases can reach surface waters (Stumm, 2004). The occurrence depends on peculiar climatic and geological conditions that allow evaporative concentration of the water (often evaporation much higher than water inputs and in endorheic basins), and on geochemical factors that favor chemical evolution towards an alkaline environment (Jones and Deocampo, 2003).

Such an example has been reported from Auckland, New Zealand, where Davidson and Black (1994) proposed that lithology strongly controlled zeolite paragenesis, with different units having different mineral assemblages. For the volcaniclastic sandstones of the Waitemata Group’s East Coast Bays Formation (ECBF), the paragenetic sequence was clinoptilolite + (mordenite) → chabazite + erionite. This sequence is low in Si and is associated with a closed hydrologic system such as a lacustrine environment, which is more alkaline than marine environments (which are more neutral; Davidson and Black, 1994). This indicates that the sandstone was likely sealed in-between layers of mudstone within the turbidite sequence, creating a closed hydrologic system. The crystallization of zeolites occurred as pore fluid flowed within the rock mass, liberating Si and alkali cations from the volcanic glass within the sandstone (Davidson and Black, 1994). Over time, the fluid composition changed as the alteration of the minerals continued, with the varying cation contents of the zeolite assemblages attributed to changes within the pore fluid chemistry (Davidson and Black, 1994; McHenry et al., 2020). In contrast, within conglomerate beds of the Waitemata Group, pore fluid was less restricted, and the system was an open hydrologic system while also highly permeable, allowing analcime to crystallize from Na-saturated fluids (Davidson and Black, 1994).

## 4 Characterization and toxicity

### 4.1 Fibrous zeolites

Most naturally occurring zeolites are non-fibrous, whereas zeolites such as clinoptilolite, edingtonite, erionite, ferrierite, gonnardite, dachiardite, kalborsite, mesolite, mordenite, natrolite, offretite, paranatrolite, scolecite and thomsonite can be fibrous (Nishido and Otsuka, 1981; Belitsky et al., 1992; Thomas and Ballantyne, 1992; Artioli and Galli, 1999; Armbruster and Gunter, 2001; Betti et al., 2022; Finocchiaro et al., 2022). In particular, epidemiological (Bariş et al., 1979; Carbone et al., 2011) and experimental data (Wagner et al., 1985; Coffin et al., 1992) show that erionite fibers have the highest carcinogenic potency among any other fibers so far studied, including fibers regulated as asbestos. Erionite fibers also have strong fibrogenic potential (Fraire et al., 1997) and biopersistence (Sanchez et al., 2009). Malignant mesothelioma (MM) is a cancer caused by a malignant transformation of the mesothelial cells which are found in the tissue lining the lungs, abdomen, and heart (Carbone et al., 2011; Carbone and Yang, 2012; Attanoos et al., 2018). Pleural MM (cancer of the tissue that lines the lungs) is the most common cancer of the three and is caused by the inhalation of fibrous material such as asbestos or erionite (Bariş et al., 1979; Robinson et al., 2005). In addition, there are also non-cancerous health issues known to be caused by inhaling erionite, such as pleural fibrosis and promoting the production of autoantibodies (Fach et al., 2003; Zebedeo et al., 2014; Ray, 2020).

### 4.2 Particle size and morphology

Particle size is one of the most critical factors determining the toxicity of a fiber. Depending on the size and morphology, the inhaled particles can be deposited in various parts of the respiratory system with very different *in situ* biochemical conditions (Giordani et al., 2019). In terms of morphology, elongated particles typically pose a higher risk to human health in comparison to spherical particles, as they are more likely to be inhaled and deposited within the lung airway surfaces (Asgharian et al., 2018). For elongated particles, NIOSH has established exposure limit guidelines for asbestos and other fibrous mineral particulates that satisfy the following size requirements: length (L) ≥5 μm and a ≥3:1 aspect ratio of length to diameter (NIOSH, 1994; Beaucham et al., 2018). The World Health Organization (WHO, 1997) also specifies a diameter (w) of <3 μm for particles to be inhalable, and fiber diameter is a critical dimension as the smaller the diameter, the higher the particulate number per unit mass of dust, which will increase the inhalation potential of the fibers (WHO, 1997). The diameter also influences the ability of phagocytes to clear fibers from the respiratory tract, and the density of the fiber aids in determining its aerodynamic diameter (d_ae_), which influences the depositional depth of fibers within the respiratory tract (WHO, 1986, 1984; James et al., 1994; Brown, 2015; DeWitt et al., 2016; Belluso et al., 2017; Gualtieri et al., 2017; Di Giuseppe, 2020). The aerodynamic diameter, as defined by Gonda and Abd El Khalik (1985) and modified by Di Giuseppe (2020), is:

$$d_{ae}=d\sqrt{\left(\frac{1}{\frac{2}{9}\left(\frac{1}{(\ln2\beta -0.5}\right)+\frac{8}{9}\left(\frac{1}{\ln2\beta +0.5}\right)}\right)\left(\frac{\rho }{\rho_{0}}\right)}$$

where *d* = fiber diameter, *β* = aspect ratio (L/w), *ρ* = particle density, and *ρ*_0_ = unit density (1 g/cm^3^; Di Giuseppe, 2020; Gualtieri et al., 2017).

The aerodynamic diameter is critical as not only can it determine where in the respiratory tract a fiber is likely to be deposited, but it also assists in determining the inhalability of a fiber (Millage et al., 2010; Gualtieri et al., 2017; Di Giuseppe, 2020). The inhalability of fibers is important as it determines if a particle may be able to enter the body (Millage et al., 2010). In terms of erionite inhalability, there is a paucity of research, with most published work focused on asbestos and other fibers (e.g., Millage et al., 2010; Shang et al., 2015; Asgharian et al., 2018; Militello et al., 2021; Vigliaturo et al., 2021; Zanko et al., 2022). Nevertheless, from these studies, the maximum inhalable dimensions of a fibrous particle have been determined. For most particulate fibers, a d_ae_ < 100 μm is considered to be inhalable, and while there exists a lack of studies of ultra-large (d_ae_ > 100 μm) particles, such particles do not pose a significant health risk due to the limited range while airborne (Millage et al., 2010; Vigliaturo et al., 2021).

Out of the 139 locations of erionite found within the literature, only 37 included information on the morphometry of the erionite fibers, and 95 provided information on the crystal habit (Supplementary Table S1), data necessary for hazard assessment. In the literature, erionite is commonly described as being elongated; however, it is not always fibrous, which is apparent by the number of varying terms used to describe the crystal habit (Van Gosen et al., 2013; Giordani et al., 2016). Terms that have been used to describe erionite include; acicular bundles (Bargar and Keith, 1984), divergent acicular aggregates (Belitskiy and Bukin, 1968), thick fibrous minerals (de Pablo-Galán and de Chávez-García, 1996), radiating clusters (Donoghue et al., 2008), hexagonal rods (Golden et al., 1993), woolly aggregates (Matassa et al., 2015), needlelike (Metropolis, 1986), compact fibrous erionite (Passaglia et al., 1974), tiny needles (Reed, 1937), lamellae in radiating aggregates (Vezzalini et al., 1994), fibrous (Hey, 1959; England and Ostwald, 1979; Birch, 1987; Van Gosen et al., 2013), hexagonal prisms (Tschernich and Wise, 1982), radiating bundles (Passaglia and Tagliavini, 1995; Saini-Eidukat and Triplett, 2014), felted masses (Surdam and Eugster, 1976), bundles of needles (Surdam and Eugster, 1976; Karakaya et al., 2015), bundles of fibrils (Dogan et al., 2006), thick bundles which cleave into blocky rods (Mumpton, 1979), acicular crystals (Deffeyes, 1959; Matsubara et al., 1978; Van Gosen et al., 2013; Giordani et al., 2016) and woolly fibers (Deffeyes, 1959; Staples and Gard, 1959; Shimazu and Mizota, 1972). Detailed descriptions of the erionite habit can be found in Supplementary Table S1.

**FIGURE 7:**
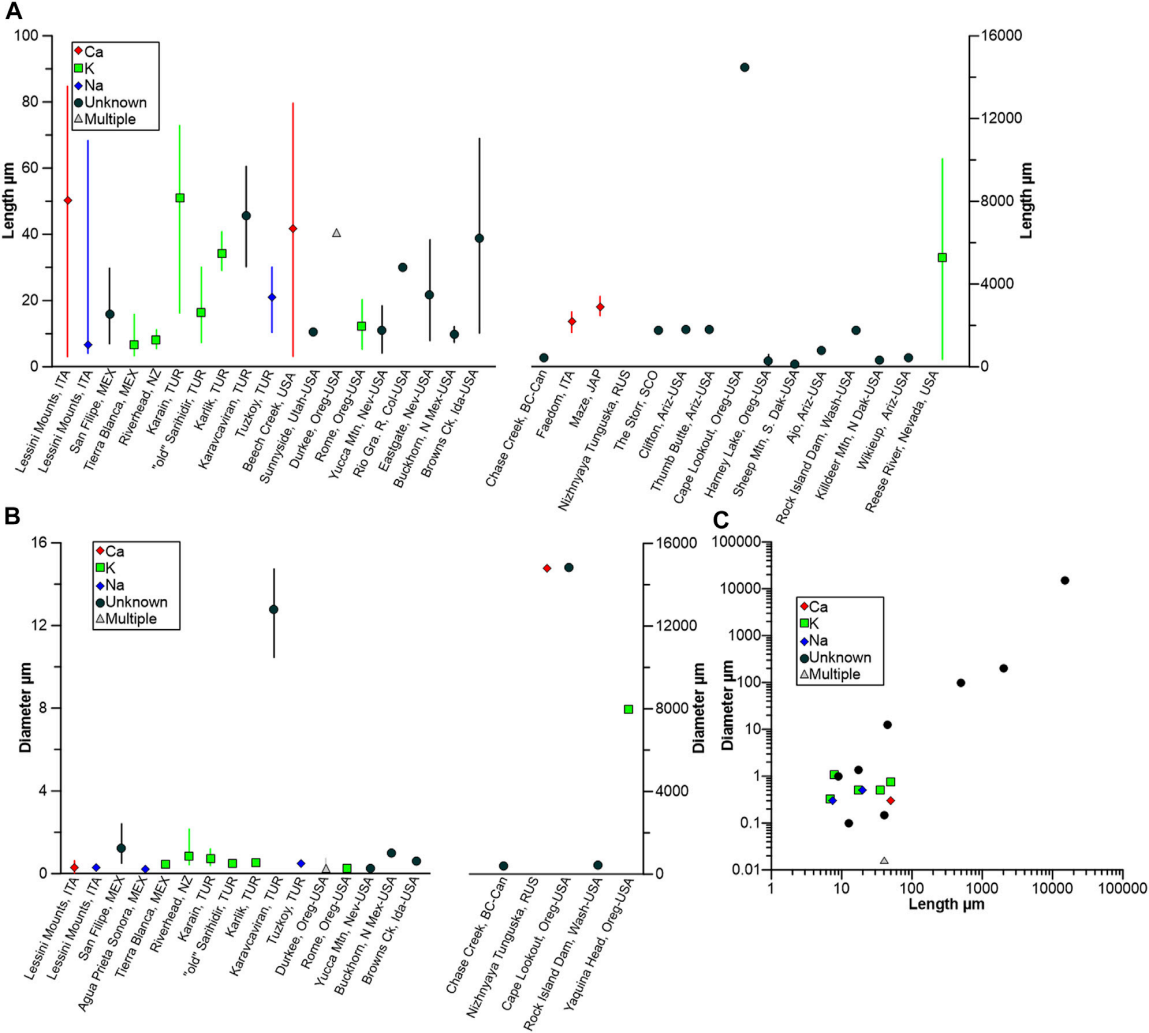
Morphometric data for erionite, coded by erionite species, where data is available: **(A)** Length of erionite fibers and location, with mean and value range (note different *y*-axes); **(B)** diameter of erionite and location, with mean and value range (note different *y*-axes); **(C)** scatterplot of length vs. diameter, coded by erionite type.

The fiber morphometric data that has also been reported in the literature is summarized in Figure 7, and a wide range of lengths (Figure 7A) have been reported, ranging from <5 μm (e.g., at Lessini Mounts, Italy Giordani et al., 2016) and at Tierra Blanca de Abajo, Mexico (Ortega-Guerrero and Carrasco-Núñez, 2014) and up to 15 mm at Cape Lookout, Oregon, United States (Wise and Tschernich, 1976; Van Gosen et al., 2013). Fiber diameters show less marked variability, with most <1 μm, irrespective of erionite series (Figure 7B). A bivariate scatterplot of length (L) and diameter (w) is shown in Figure 7C, with a moderately strong positive, linear relationship evident between the two variables. Thus, following the WHO (1997) guidelines, the data indicates that erionite could potentially be a hazard in at least 15 of the reported locations (i.e., L ≥ 5 μm, w < 3 μm, ≥3:1 aspect ratio). The rather limited morphometric dataset should be treated with caution (Figure 7C), but erionite-K may be the most likely erionite series to exhibit an inhalable morphometry.

Notwithstanding the above, potential issues exist in the reporting of erionite morphometric data (Figure 7) that may hinder inter-site comparisons. First, occasionally, the diameter of fiber bundles is reported by authors, as opposed to a single fibril, an example being the erionite morphometric data from Yaquina Head and Cape Lookout in Oregon, United States (Wise and Tschernich, 1976; Van Gosen et al., 2013). The same potential reporting issue occurs for Rock Island Dam, Washington, United States (Altaner and Grim, 1990), with uncertainty as to whether the authors measured either a single fibril or a bundle of fibrils. A second potential limitation in some of the erionite morphometric data is hinted at by the frequency distributions of fiber length. For example, the population distribution of fiber length from a single location may be bimodal rather than unimodal, and this may indicate fracturing due to handling, as at Lessini Mounts, Italy (Giordani et al., 2016). At that site, the primary mode for fiber length ranged from ∼40 to ∼60 μm, yet a secondary mode ranged from ∼10 to ∼25 μm. The secondary mode is likely linked to the fracturing of fibers, which may have occurred when collecting and preparing the samples, and therefore, incorrect natural fiber lengths may then be reported by authors. A compounding issue is then using the sample mean to represent a bimodal distribution (Riffenburgh, 2012), when the means and standard deviations of each mode, along with a mixing parameter, should usually apply (e.g., Ashman et al., 1994).

### 4.3 Biopersistence

Biopersistence is the amount of time the mineral fibers reside within the human body following inhalation, and fibers that cannot be cleared from the respiratory tract are considered biopersistent and can therefore accumulate during chronic exposure (e.g., Bernstein et al., 2005). Erionite fibers exhibit biopersistence, and erionite-induced mesotheliomas have similar histology and long latency to those originating from asbestos, though there are still uncertainties in their respective mechanisms of carcinogenicity (Reid et al., 2021). Two key components of erionite biopersistence are 1) biodurability and 2) dissolution in surfactant or physiological fluids (Moolgavkar et al., 2001). Regarding biodurability, longer, asbestiform fibers tend to exhibit high tensile strength and elasticity (Giordani et al., 2017). In addition, *in vitro* acellular dissolution studies have demonstrated that while chrysotile dissolves faster than amphibole asbestos, Scholze and Conradt (1987) showed that erionite is much more biopersistent than both crocidolite and chrysotile. This is consistent with the reported mineral fiber dissolution rates reported by Gualtieri et al. (2018). For a 0.25 μm thick fiber, the calculated dissolution time of chrysotile is ∼94–177 days, very short if compared to that of amphibole fibers (49–245 years) and fibrous erionite (181 years). Thus, the biopersistence of erionite is important because the fiber can induce carcinogenicity only if it is durable enough to remain physically and chemically intact within lung tissue (Sanchez et al., 2009).

### 4.4 Iron and erionite

In addition to needle-like particle morphology and biopersistence, a key factor contributing to the toxicity potential of erionite has historically been deemed to be the presence of iron (e.g., Fubini and Mollo, 1995). Indeed, it is believed that the toxicity of erionite is linked to both its fibrous properties and its association with iron in natural deposits (Fach et al., 2003; Sanchez et al., 2009; Reid et al., 2021). One theory is that the toxicity of erionite is associated with iron that accumulates on the surface of the fibers and generates cytotoxic hydroxyl radicals (Fach et al., 2003; Waris and Ahsan, 2006; Pacella et al., 2018). Fraire et al. (1997) reported that erionite from Rome, Oregon, and Pine Valley, Nevada, shows contrasting effects *in vivo*. The sample from Rome is Fe-rich, whereas the sample from Pine Valley is Fe-poor, and results showed that Rome erionite, with Fe in some form, is more potent than Fe-poor erionite (Fraire et al., 1997). It is thought that iron in mineral fibers may be responsible for carcinogenic activity, namely *via* reactive oxygen species (ROS) or reactive nitrogen species (RNS) production (Roggli et al., 2010; Pacella et al., 2018). Active iron present at the surface of the fibers promotes the formation of reactive hydroxy species by the surface Fenton reaction chain (Gualtieri et al., 2018). However, the presence of iron in fibrous erionite is currently debated, and experiments by Gualtieri et al. (2018) concluded that erionite fibers may not in fact, contain structural Fe^3+^, but contain Fe^3+^ associated iron-rich impurities. Indeed, Gualtieri et al. (2016) showed that Fe found in some erionite analyses was actually coming from iron-bearing nano-particles on the surface of the erionite fibers. The fact that iron is not found in the erionite crystal structure of natural samples also has a sound geological basis. This is because, during the zeolitization process, iron typically present as Fe^2+^ in the host tuffs is leached, oxidized, and precipitated later as secondary iron-bearing phases like iron hydroxides (Gualtieri et al., 2018).

## 5 Erionite analysis and identification

As highlighted above, erionite pathogenicity can be related to a number of physico-chemical properties (Pacella et al., 2017; Gualtieri et al., 2018; Carbone et al., 2019). Therefore, delineating the chemistry as well as the surface characterization of the involved particle is important (Giordani et al., 2022). However, since erionite was first discovered, a multitude of studies have been conducted globally that involve classifying erionite, yet the precise identification of erionite has been somewhat hampered by its physico-chemical similarities to other fibrous zeolites such as offretite (Passaglia et al., 1998; Dogan and Dogan, 2008; Ray, 2020). Erionite has not been mined for commercial use since the late 1980s (Ray, 2020), and so many commercial laboratories focusing on asbestos in bulk building materials are inexperienced in identifying erionite. A related issue is that in contrast to the regulated asbestos minerals, erionite mineral fibers do not have established occupational exposure limits (OELs), so specific analytical methods and approaches are somewhat lacking.

### 5.1 Sample preparation

**FIGURE 8:**
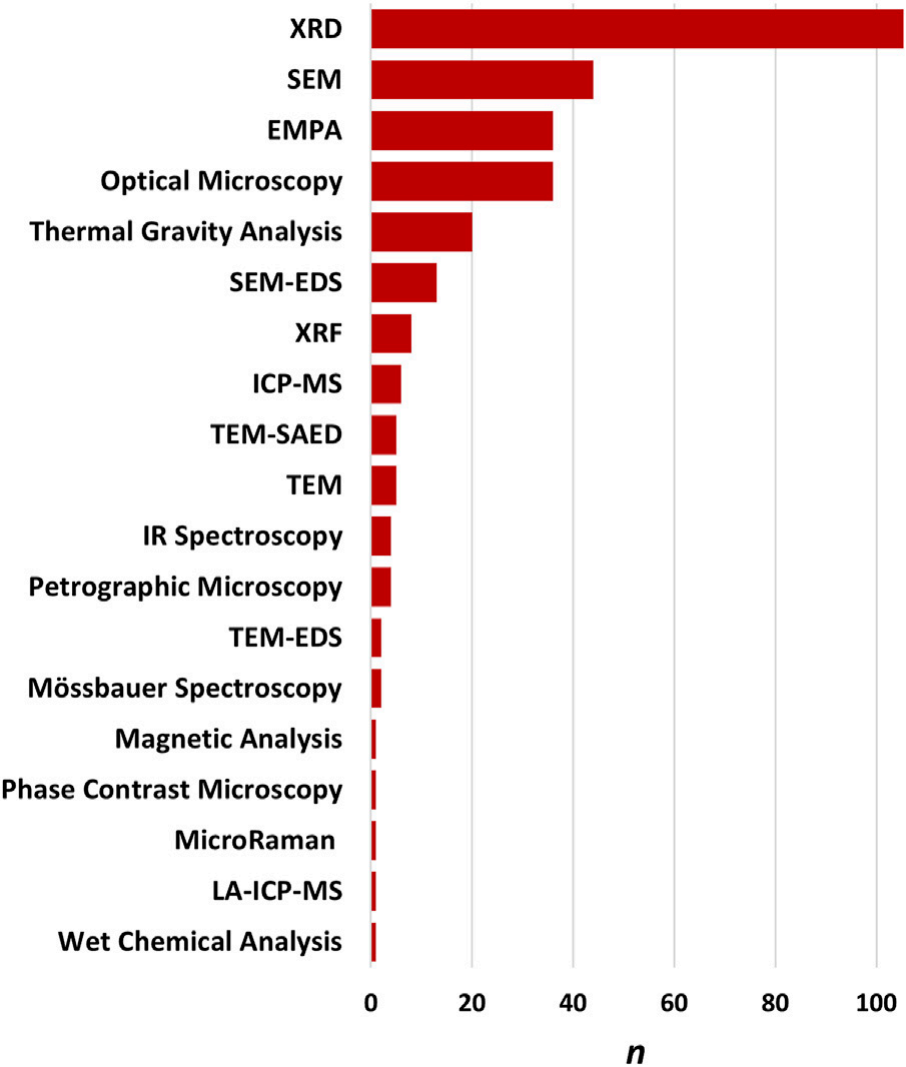
Frequency of each method used to identify and characterize erionite. XRD is the most frequent, followed by SEM and EMPA.

A range of techniques have been used over the decades to study erionite (Figures 2, 8). These can be generally grouped into techniques used for 1) bulk mineral analysis or 2) techniques for analyses of single mineral fibers (Dogan and Dogan, 2008). Bulk mineral analysis techniques that have typically been applied to the study of erionite include X-Ray Diffraction (XRD), Inductively Coupled Plasma Mass Spectrometry (ICP-MS), and X-ray fluorescence (XRF). Single mineral fiber analysis approaches include Polarized Light Microscopy (PLM), Phase Contrast Microscopy (PCM), Scanning Electron Microscopy with Energy Dispersive Spectroscopy (SEM-EDS), Transmission Electron Microscopy with Energy Dispersive Spectroscopy (TEM-EDS), and Electron Micro Probe Analysis (EMPA). Some example methods and case studies are discussed in the following sections. Screening level analysis of erionite, using PLM is occasionally used but requires high-dispersion refractive index liquids with the appropriate refractive index range (*n* = 1.460 to 1.480; Berry et al., 2019). PCM has also been used infrequently for screening of soils for erionite. An example is the work of Farcas et al. (2017), who recently reported the use of PCM analysis for detecting erionite from soils sampled in eastern Montana and western South Dakota. Farcas et al. (2017) compared the PCM analysis to PLM analysis of erionite in soils and found the PCM method to be more sensitive than PLM.

Binocular microscopy, PLM, and PCM are unreliable when discriminating amongst different fibrous minerals, such as erionite, offretite, or asbestos fibers, but may be helpful to determine if fibrous minerals are present within a sample (e.g., Berry et al., 2019). However, irrespective of the techniques utilized, Ray (2020) cautioned that erionite is more delicate to handle than asbestos minerals when preparing samples for analysis. For example, rock and soil material must be reduced to a fine powder for analysis by optical microscopy (OM), and based on the preparation of erionite samples from Pine Valley, Nevada, Ray (2020) reported that erionite is fragile and extremely susceptible to over grinding. Indeed, samples were milled to two nominal sizes, 250 μm and 75 μm, and once over-milled, bundles and fibers were destroyed and broken into non-fibrous particles (Ray, 2020). Thus, such fragments would no longer be countable by an analyst during a microscopic examination, which could misrepresent the potential toxicity of *in situ* erionite material. For the detection of erionite fibers in soils, the fluidized bed asbestos segregator (FBAS) preparation method is often used for both asbestos and erionite fibers (e.g., Berry et al., 2019). In particular, previous research has demonstrated that using an FBAS, even very low levels (0.002%–0.005% by weight) of fibers in soils can be readily detected when followed by TEM (Januch et al., 2013).

### 5.2 X-ray diffraction

X-ray powder diffraction (XRD) is a convenient technique that can reveal detailed structural and chemical information about the crystallography of the material. The information XRD provides is especially advantageous as XRD can analyze the constituents of a bulk sample of heterogeneous rock. The likely minerals present within the sample can potentially be identified from the XRD diffraction patterns using online databases and software developed for XRD. The presence of erionite within bulk rock samples has been identified, which makes it a particularly valuable tool (Beaucham et al., 2018). Bulk XRD will not only provide detailed mineralogy of the composition of rock samples but also identify the different zeolites in a given specimen *via* their XRD pattern. However, there are fundamental issues in applying XRD on its own. For example, erionite and offretite can occur together and exhibit very similar XRD patterns, and second, low concentrations of erionite may be masked by diffractions from other minerals (Dogan and Dogan, 2008). Thus, XRD should be applied in combination with other methods, such as SEM-EDS, where individual fibers or fiber bundles can also be imaged. A further limitation of XRD is that the minimum amount of mineral needed to be present within the sample is 1%–2%, as any concentrations below this threshold will not be detected (Meeker, 2008; Eylands et al., 2009).

### 5.3 X-ray fluorescence

X-ray fluorescence (XRF) spectroscopy is a technique also used to analyze samples to determine their chemical composition. It is similar to EMPA, however, it is not as precise and is typically used for bulk rock analysis (Dogan and Dogan, 2008; Stocker et al., 2017; Oyedotun, 2018). XRF works by using X-rays to excite atoms, which causes electrons to be dislodged from the inner orbital, producing fluorescent radiation (Oyedotun, 2018). The energy of the photons emitted is distinct for the transition between specific electron orbitals within an element, and it can be measured and used to determine the abundance of the elements present within the sample being studied (Oyedotun, 2018).

### 5.4 Inductively coupled plasma mass spectrometry

As outlined above, computing the balance error (E%), the K-content, and the Mg-content is fundamentally important for accurate characterization of erionite, and ICP-MS is routinely used for this purpose (e.g., Dogan and Dogan, 2008). ICP-MS is also used to verify putative erionite detected that has been tentatively identified using other methods, such as SEM-EDS (e.g., Dogan and Dogan, 2008). ICP-MS has also been used to identify trace elements present on erionite fibers that may also play a role in fiber toxicity (e.g., Bloise et al., 2016), as well as studying the possible uptake of arsenite and arsenate (H_2_AsO_4_) species from aqueous solution in zeolites including erionite (Elizalde-González et al., 2001).

### 5.5 Scanning electron microscopy-energy dispersive spectroscopy

Given the limitations of some of the bulk analysis approaches outlined above, SEM-EDS can provide improved delineation of single minerals within a sample. SEM involves scanning an electron beam over a sample to create an image. The images of rock specimens can provide detailed information on the morphology of minerals, which is an advantageous technique when looking specifically for fibrous zeolites such as erionite (Giordani et al., 2017). Numerous studies have utilized SEM to identify erionite (Pacella et al., 2016; Giordani et al., 2017; Rinaudo and Croce, 2019). These authors used SEM primarily due to the ease at which fibers can be identified within the analyzed samples. For minerals that may look similar, EDS can be used to distinguish the minerals from one another. Additionally, SEM can image the minerals in their natural habitat for freshly fractured samples. Thus, not only can the zeolites themselves be observed, but so can the minerals that surround them. The images can provide details of the zeolite facies mineral assemblage, aiding a better understanding of the zeolites.

Energy Dispersive Spectroscopy (EDS) is a technique used in conjunction with electron microscopy (SEM or TEM). When the beam of electrons hits a sample, it generates X-rays, which are characteristic of each element (Ray, 2020). The EDS detects the X-ray energy and measures the rate at which the X-ray is emitted, producing an EDS spectrum of X-ray energy vs. counts. The spectrum gives the elemental composition of the selected sample. In this way, EDS works to quantify and identify every element within the periodic table except H, Li, and He (Newbury and Ritchie, 2013). Aside from being unable to identify every element within the periodic table, there are other areas where EDS will be imprecise. One of these areas is using EDS to measure an object where the geometry varies, as this can introduce a geometric error that can alter the quantitative results to the point that they become useless (Newbury and Ritchie, 2013). Another limitation is that during EDS analysis, the electron beam has been found to replace cations in prior mineral studies, causing the fibers to become unstable, especially if their diameter is <0.5 μm (Carbone et al., 2011; Pacella et al., 2016; Ray, 2020). This is the case for both SEM and TEM-EDS (Carbone et al., 2011; Pacella et al., 2016; Ray, 2020). Precautions to minimize the error as discussed by Pacella et al. (2016) should be taken into account. For example, when determining the chemical composition, calculating the correction factor for each oxide as a function of its particle size is useful. This will reduce size-dependent errors from arising, especially for smaller particles.

### 5.6 Transmission electron microscopy

Transmission electron microscopy (TEM) accelerates a beam of electrons through a sample prepared on a small grid to observe small specimens’ morphology and structure. According to Van Gosen et al. (2013) and Ray (2020), some techniques using electron beams, such as TEM, can be less effective on zeolites, as the beam can influence the chemistry and crystal structure of the mineral. While asbestos fibers tend to be thermally stable (Martin et al., 2016), most zeolites are quite sensitive to the electron beam (Ray, 2020). Once the energy of the electron beam collides with the erionite fibers, they deform (Ray, 2020). This degradation caused by the electron beam also influences the chemistry and crystal structure. Indeed in standard TEM analyses of erionite, the selected area electron diffraction (SAED) pattern does not last long enough to be documented and measured to an appropriate standard (Van Gosen et al., 2013; Ray, 2020). As mentioned above electron beams have been found to replace some cations in prior studies of zeolites, especially during EDS analysis and the key to overcoming these problems is stabilization using cryogenic electron microscopy holders (Carbone et al., 2011; Ray, 2020). The cryogenic holders aid in stabilizing zeolite fibers during TEM, protecting them from the energy of the electron beam (Gualtieri et al., 1998; Ray, 2020). The cryogenic holder is simply the addition of cooling by liquid nitrogen. In addition, Ray (2020) has also drawn attention to the issue of over-milling with regard to TEM, which could degrade the natural morphology of the erionite mineral prior to analysis.

### 5.7 Electron micro probe analyzer

Similar to TEM and SEM, EMPA utilizes an electron beam to bombard a solid material and determine the sample chemistry, and can be used on geological materials *in situ* to acquire data which is quantitative and precise (∼1 μm; Reed, 2005). However, unlike SEM, EMPA requires a smooth polished flat surface for analysis to prevent the imperfections on the surface from interfering with the sample and electron beam interactions (Richter and Mayer, 2012). Limitations of EMPA include the fact that lighter elements cannot be detected, such as hydrogen and helium (Bennell, 2015). Furthermore, similar to EDS, the electron beam may cause the migration of cations away from the beam. Indeed, the movement of alkalis (especially Na) can also cause the concentration of Si + Al to increase, affecting the quantitative determination of the mineral composition (Kearns and Buse, 2012; Campbell et al., 2016). To decrease these effects, Campbell et al. (2016) have recommended operating protocols to determine zeolite compositions, including using a smaller defocused beam with a diameter of 20 μm, as well as prioritizing detection of elements such as Na, K, and Al with the spectrometer configuration first. The chemical data should also be obtained from several individual point analyses on each sample, to determine the homogeneity of the mineral and account for possible cation migration (Campbell et al., 2016). Additionally, as EMPA reports chemical data as oxides of the different elements, further calculations need to take place to determine the mineral formulae (Kearns and Wade, 2021). When the mineral composition has been determined, the results should be evaluated against the charge balance error formula (E%; Dogan and Dogan, 2008; Passaglia, 1970), Mg content test (Dogan and Dogan, 2008), and the K content test (Cametti et al., 2013).

### 5.8 Raman spectroscopy

Raman spectroscopy is a qualitative and quantitative technique involving shining a monochromatic laser beam on a sample (Bumbrah and Sharma, 2016), and several erionite studies have utilized the method (e.g., Croce et al., 2013). Typically, for different mineral species, spectral ranges of 4,000–100 cm^−1^ are recorded (Rinaudo and Croce, 2019). The resulting interaction between the laser and the atoms within the specimen causes the light to scatter, and a fraction of the scattered light changes color (Bumbrah and Sharma, 2016). The changing color is due to a change in frequency caused by energy interacting with molecular vibrations. Raman spectroscopy studies the vibration of atoms to provide information on the chemical structure, phase, crystallinity, and the material’s composition, as each mineral has a unique Raman frequency (Lancelot, 2010). Minimal sample preparation is required for Raman spectroscopy, reducing the chances of sample loss and helping to ensure the original shape of the minerals remains intact (Croce et al., 2015).

In micro-Raman spectroscopy, the laser beam is focused through a microscope. This allows the diameter of the sample being analyzed to be as small as ∼200 nm, and it thus increases the precision when determining where in a sample the laser should be directed for analysis (Kattumenu et al., 2012; Piccardo et al., 2013). Micro-Raman spectroscopy has been applied to samples of erionite from different localities in Oregon and North Dakota (United States) and Cappadocia (Turkey) by Rinaudo and Croce (2019). Rinaudo and Croce (2019) also reported that the technique can be used to observe material lying on top of the mineral structure, which cannot be observed with other analytical techniques. Micro-Raman has also been applied to the study of erionite within various organs (pancreas, spleen, and liver) of mice injected with erionite (Croce et al., 2013). Indeed, Croce et al. (2013) showed that micro-Raman spectroscopy permits the recording of distinct Raman patterns of both crocidolite and erionite fibers in animal tissues and human biopsies, so it is useful in determining fiber exposure of MM patients (i.e., erionite, crocidolite, etc.).

### 5.9 Misidentification of erionite

Dogan and Dogan, (2008) provided a seminal review on putative erionite, and reported the probability of erionite being wrongly identified by several authors in the past. This was based on re-analysis, particularly using ICP-MS, to determine if the erionite balance error (E%) is ≤ 10% (Passaglia, 1970; Dogan and Dogan, 2008). The Mg^2+^ content must also not exceed 0.80 atoms per cell, and if it does, the mineral should not be characterized as erionite (e.g., Gualtieri et al., 1998; Dogan and Dogan, 2008). Indeed, Dogan and Dogan, (2008) reported ambiguous definitions, incorrect identifications, and inaccurate reporting of clinical investigations in their review. Moreover, Dogan and Dogan (2008, p. 355) concluded that “if data did not pass either the E% or Mg content test, then we propose that reference to them in the literature be disregarded.”

Given the above stringent reporting caveats stated by Dogan and Dogan, (2008), prior to 2008, the typical erionite identification issues were threefold: 1) erionite was sometimes often confused with offretite (e.g., Birch, 1988); 2) the exact erionite species was not reported (Sheppard and Gude, 1969); or 3), the erionite species was reported, but was incorrect (e.g., Dogan and Dogan, 2008). For example, Sheppard et al. (1974) utilized XRD to originally identify a zeolite overgrowth on levyne as offretite at Beech Creek, United States. Subsequent analysis by Passaglia et al. (1998) found the sample was erionite, based on XRD and EMPA, as well as other techniques. The frequent misidentifications of erionite as offretite have been due to the similar morphologies and crystal structure of these two zeolites. In some cases, despite the reporting of the chemical formula, confusion can still occur, such as the zeolite from Araules, Loire, France. This was initially identified as offretite by Pongiluppi (1976) and subsequently re-identified as erionite by Passaglia et al. (1998). However, Gualtieri et al. (1998) then determined that the mineral belonged on the erionite-offretite border, with no explicit characterization of what it could be. Finally, Dogan and Dogan (2008) classified the mineral as not being erionite because the zeolite sample had an Mg content of 0.83, which did not meet the requirement of Mg < 0.80 to be classified as erionite.

## 6 Future research directions and conclusion

This review has determined that erionite is found globally in a host of different geological environments. From the morphological data reported, it would appear that all end-member species of erionite (Ca, K, Na) can potentially be inhalable. Erionite is most commonly found in rocks from the Miocene epoch (23–5.3 Ma), although it is also present in some 1.90–1.87 Ga rock units formed during the Pre-Cambrian, to the Pleistocene (<2.58 Ma). Nevertheless, the exact age of zeolitization within the host rocks is usually unknown, but obviously, post-dates rock formation. The typical host rocks for erionite are mafic and felsic, primarily basalt and tuff, respectively. Within tuff/felsic rocks, erionite commonly is within the matrix, and within basalt/mafic rocks, erionite is found in vesicles.

A clear goal in the future is to determine which analytical techniques are the most suited to delineate erionite from other zeolites in order to prevent future misidentification of erionite. Previous work has recommended techniques for both bulk sample analysis and singular mineral analysis (Dogan and Dogan, 2008; Ray, 2020). Given that erionite has two key characteristics of interest regarding toxicity, namely 1) chemistry and 2) morphology, it is important that whatever analytical approaches are implemented, quantitative chemistry and accurate particle morphology can be reported. While SEM-EDS is a key technique in analyzing zeolite fibers, most recently, cryogenic TEM-EDS in conjunction with ICP-MS have been shown to have the potential to be the most accurate. Fundamentally, after the chemical composition is determined, it is prudent to calculate the Mg content, K content and the balance error (E%) to reliably establish if the mineral is actually erionite, or not. Future research could focus on several areas. First, accurate dating of when the host rocks were zeolitized may help in delineating the geographic distribution of erionite-bearing units. Second, analysis of associated zeolites would assist in determining the paragenetic sequence of formation, which is useful for geological studies. Third, the effects of preparation methods on the morphology of erionite fibers should be explored, and optimum milling protocols could be identified to enhance the replicability of results. Finally, major differences between erionites from magmatic vesicles and those that have crystallized in volcaniclastic sedimentary rocks need to be fully explored in terms of toxicity and hazard. A better understanding of where erionite forms and occurs is a key to robust risk assessments, and the establishment of effective mitigative measures to prevent future exposure to erionite.
