# Supplementary material for: Global geological occurrence and character of the carcinogenic zeolite mineral, erionite: A review
Source: Front Chem. 2022 Nov 18;10:1066565. doi: 10.3389/fchem.2022.1066565 (PMC9715606; doi:10.3389/fchem.2022.1066565)
Supplement: Supplementary file 1 [file Table1.DOCX]

Supplementary Table 1: Summary of available erionite information from published sources, with analytical method abbreviations described in-text, listed alphabetically by country (where possible). Abbreviations are as follows: *ana* = analcime, *bog* = boggsite, *chab* = chabazite, *clin* = clinoptilolite, *cowl* = cowlesite, dach = dachiardite = epistilbite, *fauj* = faujasite, *ferr* = ferrierite, *garr* = garronite, *gmel* = gmelinite, *got* = gottardite, *harm* = harmotome, *heu* = heulandite, *lev* = levyne, *meso* = mesolite, *mord* = mordenite, *natr* = natrolite, *off* = offretite, *paul* = paulingite, *phil* = phillipsite, *scol* = scolecite, *stil* = stilbite, *thom* = thomsonite, *tsch* = tschernichite, *wil* = wilhendersonite, *yug* = yugawaralite.

| Location | Type | Habit | Formation/ Environment | Other zeolites | Methods | Source |
| --- | --- | --- | --- | --- | --- | --- |
| Campbell Glacier, Antarctica | Na | fine radiate aggregates of yellowish-orange fibres | hydrothermal alteration | heu, mord | bi microscope, XRD, XRF, EMPA, OM, TGA | (Dogan and Dogan, 2008; Passaglia et al., 1998; Vezzalini et al., 1994) |
| Mount Adamson, Antarctica | Na | transparent lamellae in radiating aggregates, overgrown on levyne | hydrothermal alteration | heu, lev, thom, scol, epi, mord, phil, stil, tsch, bog, cowl, got | bi microscope, XRD, XRF, EMPA, Optical, TGA | (Dogan and Dogan, 2008; Passaglia et al., 1998; Vezzalini et al., 1994) |
| Cairns Bay, Australia | Na | n/a | hydrothermal alteration | ana, natr, gmel, chab, cowl, lev, phil, thom | OM, XRD, EMPA, TGA | (Birch, 1988; Dogan and Dogan, 2008; Passaglia et al., 1998) |
| Jindivick, Australia | Ca | fibrous | n/a | chab, lev, thom, phil, ana | OM, XRD, EMPA, TGA | (Birch, 1987; Dogan and Dogan, 2008; Passaglia et al., 1998) |
| Merriwa, NSW, Australia | Ca | fibrous | n/a | lev, chab | OM, XRD, EMPA, TGA | (Dogan and Dogan, 2008; England and Ostwald, 1979; Passaglia et al., 1998) |
| Phillip Island, Australia | Ca, Na | n/a | hydrothermal alteration | ana, chab, ferr, gmel, heu, lev, natr, phil, stil, thom | XRD, EMPA, TGA, OM | (Birch, 1988; Dogan and Dogan, 2008; Passaglia et al., 1998) |
| Sankt Paul im Lavanttal, Austria | n/a | n/a | n/a | n/a | OM, SEM | (Waltinger and Zirkl, 1974; Zirkl et al., 1962) |
| Eastern Rhodopes, South Bulgaria | n/a | n/a | low T° hydrothermal alteration/diagenesis in a closed system | mord, clin ana, stil | XRD, PLM, SEM, EMPA, TGA | (Ivanova et al., 2001; Kirov et al., 2011) |
| Chase Creek, British Columbia, Canada | n/a | colourless to golden-yellow hexagonal prisms | n/a | paul | EMPA, SEM | (Tschernich and Wise, 1982) |
| Westwold, British Columbia, Canada | Ca | erionite overgrowth on levyne | n/a | lev | EMPA, XRD, SEM, TGA, OM | (Dogan and Dogan, 2008; Passaglia et al., 1998; Wise and Tschernich, 1976) |
| Gran Canaria, Canary Islands | n/a | fibrous, radiating cluster | hydrothermal alteration in an epithermal system | n/a | XRD, SEM, XRF | (Donoghue et al., 2008) |
| Karadag Reserve, Crimea | n/a | n/a | n/a | n/a | n/a | (Suprychev and Prokhorov, 1986) |
| Prackovice nad Labem, Czech Republic | n/a | n/a | hydrothermal alteration | offr | OM, XRD, IR, Thermal Analysis, SEM-EDX | (Rychly et al., 1982) |
| Faroe Islands, Denmark | n/a | fibrous | n/a | chab | XRD | (Hey, 1959) |
| Barotu, Fiji | n/a | prismatic crystal | n/a | n/a | SEM | (Ram et al., 2019) |
| Lake Lappajärvi, Finland | n/a | colourless fibres | hydrothermal alteration | heu, stil, chab, mord | XRD | (Lehtinen, 1976; Schmieder and Jourdan, 2013) |
| Shourdo, Georgia | Ca | white - faintly yellow divergent accumulations/loose fibrous masses | n/a | chab, mord, stil | OM, XRD, EMPA, TGA | (Batiashvili and Gvakharia, 1968; Dogan and Dogan, 2008; Gualtieri et al., 1998; Passaglia et al., 1998) |
| Hvalstod Hvalfjordur, Iceland | n/a | long needle-like crystals | n/a | n/a | n/a | (Metropolis, 1986; Tschernich, 1992) |
| Kandovan, Iran | n/a | n/a | high alkaline and pH | n/a | n/a | (Ilgren et al., 2015) |
| Faedo, Vicenza, Italy | Ca | clear-white radiating bundles of needles | dissolution of limestone at intrusive contact | chab, heu, mord, stil, thom | OM, SEM, XRD, EMPA, TGA | (Dogan and Dogan, 2008; Passaglia et al., 1998; Passaglia and Tagliavini, 1995) |
| Lessini Mounts, Italy | Ca, Na | radial aggregates of prismatic crystals, single acicular crystals, radial aggregates of acicular to fibrous crystals; fibrous crystals of intergrowing levyne and erionite | hydrothermal alteration in vesicles and veins of submarine volcanics | anal, natr, heu, stil, chab, phil, harm, gmel, wil, yug, lev | SEM-EDS, EMPA, XRD | (Giordani et al., 2017, 2016; Mattioli et al., 2016) |
| Nuoro, Sardinia, Italy | Ca | compact fibrous erionite intergrowth with levyne | n/a | lev | OM, EMPA, XRD, SEM, TGA | (Dogan and Dogan, 2008; Passaglia et al., 1998, 1974) |
| Ashio, Tochigi Prefecture, Japan | n/a | acicular crystals | high vapour pressure conditions | phil, clin | OM, XRD, SEM, EMPA | (Matsubara et al., 1978) |
| Chojabaru, Iki Island, Japan | n/a | woolly fibres | diagensis | lev, stil | OM, EMPA, XRD | (Shimazu and Mizota, 1972) |
| Maze, Japan | Ca | woolly white radial aggregates - needle like appearance | n/a | heu, chab | OM, IR, XRD, Wet chemical analysis, EMPA | (Dogan, 2003; Harada et al., 1967; Kawahara, 1967; Schlenker et al., 1977; Shimazu and Kawakami, 1967) |
| Lake Magadi, Kenya | n/a | felted masses and bundles of needles | diagenesis in a saline lake fed by alkaline hot springs | ana, chab, clin | XRD, SEM, EMPA | (Bernhart Owen et al., 2019; Hay, 1964; Surdam and Eugster, 1976) |
| Oloronga Beds, Kenya | n/a | felted masses and bundles of needles | diagenesis in a saline lake fed by alkaline hot springs | ana, clin, chab, phil, mord, | XRD, SEM, EMPA | (Bernhart Owen et al., 2019; Surdam and Eugster, 1976) |
| Agua Prieta Sonora, Mexico | Na | compact elongated prisms. | diagenesis in saline lake | chab, clin, heu | OM, SEM-EDX, XRD, EMPA, TGA | (Cochemé et al., 1996; García-Sosa and Solache Ríos, 1997; Quiroz-Estrada et al., 2020, 2019) |
| Guanajuato, Mexico | n/a | fibrous | diagenesis via hydrolysis in open hydrologic system | clin, mord | OM, XRD, SEM-EDX, IR | (de Pablo-Galán and de Chávez-García, 1996) |
| San Filipe, Guanajuato, Mexico | n/a | flexible hair like and intact needle like crystals | diagenesis and low-grade metamorphism | clin, mord, chab | PLM, XRF-ED, LAICPMS, XRD, SEM-EDS | (Kshirsagar et al., 2021) |
| Tierra Blanca, Guanajuato, Mexico | K | acicular fibres / fibre bundles | diagenesis in slightly thermal, basic groundwater conditions | heu, clin | PLM, SEM-EDS, XRD, XRF, μRaman Spectroscopy, TEM-EDS, TEM-SAED | (Ortega-Guerrero et al., 2015; Ortega-Guerrero and Carrasco-Núñez, 2014) |
| Kaipara, New Zealand | n/a | acicular bundles | hot-spring activity accompanied hydrothermal alteration at shallow burial depth | chab. clin | XRD, SEM | (Sameshima, 1978) |
| TVZ, New Zealand | n/a | n/a | n/a | n/a | n/a | (Irwin, 2016) |
| Moeraki, Otago, New Zealand | n/a | n/a | n/a | ana, chab, clin, phil | n/a | (Anthony et al., 2003) |
| TVZ, New Zealand | n/a | needles | silica sinter dissolution, hydrothermal alteration | n/a | SEM, XRD | (Rodgers et al., 2004) |
| Riverhead, New Zealand | K, Na | acicular hexagonal bundles | diagenesis in closed hydrologic system within turbidites | clin, mord, chab, ana | XRD, SEM-EDS, TEM | (Davidson and Black, 1994; Patel and Brook, 2021) |
| Umukuri, Waikato, New Zealand | n/a | n/a | n/a | n/a | XRD | (Campbell et al., 2001) |
| Ballyclare N. Ireland | Ca | n/a | n/a | lev | OM, EMPA, XRD, TGA | (Dogan and Dogan, 2008; Passaglia et al., 1998) |
| Bog Hill Quarry, N. Ireland | Ca | n/a | n/a | n/a | OM, EMPA, XRD, TGA, | (Dogan and Dogan, 2008; Passaglia et al., 1998) |
| Dunseveric, N. Ireland | Na | n/a | n/a | lev | OM, EMPA, XRD, TGA | (Dogan and Dogan, 2008; Passaglia et al., 1998) |
| Lady Hill Quarry, N. Ireland | Ca | n/a | n/a | lev | OM, EMPA, XRD, TEM, TGA | (Dogan and Dogan, 2008; Gualtieri et al., 1998; Passaglia et al., 1998) |
| Nizhnyaya Tunguska, Russia | Ca | monomineralic divergent acicular aggregates, fresh = pale green; weathered = white | n/a | heu | OM, XRD, EMPA, TGA | (Alberti et al., 1997; Belitskiy and Bukin, 1968; Dogan and Dogan, 2008; Gualtieri et al., 1998; Passaglia et al., 1998) |
| Isle of Skye, Scotland | Ca | n/a | n/a | lev | OM, EMPA, XRD, TGA | (Dogan and Dogan, 2008; Passaglia et al., 1998) |
| Isle of Skye, Scotland | n/a | platy-fibrous crystal aggregates | n/a | garr | XRD | (Macpherson and Livingstone, 1982) |
| Yeongil Area, South Korea | n/a | micro-crystalline aggregates, hexagonal needle shaped | dFbiagenesis | clin, heu, mord, fer, chab, ana, meso, natr | PLM, XRD, SEM, EMPA | (Noh and Kim, 1986) |
| Olduvai Gorge, Tanzania | K, Na | n/a | diagenesis in saline alkaline lake | phil, natr, chab, ana | XRD | (Hay, 1964, 1963; McHenry et al., 2020) |
| "old" Sarihidir, Turkey | K | individual fibres | lacustrine, high alkalinity | clin, chab, ana | OM, XRD, SEM-EDS, ICP-MS, TEM-SAED, XRF | (Birsoy, 2002; Dogan, 2003; Dogan et al., 2006; Dogan and Dogan, 2008; Mumpton, 1979; Temel and Gündoğdu, 1996) |
| Karain, Turkey | K | bundles of needles | lacustrine, high alkalinity | clin, chab, mord | OM, XRD, SEM-EDS, ICP-MS, TEM-SAED, XRF | (Birsoy, 2002; Dogan, 2003; Dogan et al., 2006; Dogan and Dogan, 2008; Mumpton, 1979; Temel and Gündoğdu, 1996) |
| Karavcaviran, Turkey | n/a | thick bundles which cleave into blocky rods | lacustrine | n/a | XRD, SEM | (Mumpton, 1979) |
| Karlik, Turkey | K | bundles of individual fibrils | lacustrine, high alkalinity | n/a | OM, XRD, SEM-EDS, ICP-MS, TEM-SAED, XRF | (Birsoy, 2002; Dogan, 2003; Dogan et al., 2006; Dogan and Dogan, 2008; Mumpton, 1979; Temel and Gündoğdu, 1996) |
| Tuzgölü Basin, Turkey | n/a | Bundles of fibrous and needle shaped crystals | diagenetic alteration in a closed system, within a saline alkaline lake | heu, clin, chab, ana | OM, XRD, SEM, ICP-MS | (Karakaya et al., 2015) |
| Tuzkoy, Turkey | Na | individual fibres / bundles of needles | lacustrine, high alkalinity | clin, chab, ana | OM, XRD, SEM-EDS, ICP-MS, TEM-SAED, XRF | (Birsoy, 2002; Dogan, 2003; Dogan et al., 2006; Dogan and Dogan, 2008; Mumpton, 1979; Temel and Gündoğdu, 1996) |
| Ajo, Pima County, AZ, USA | n/a | hexagonal needles and radial groups | n/a | n/a | n/a | (Van Gosen et al., 2013) |
| Bear Springs, AZ, USA | n/a | n/a | n/a | chab, phil | XRD | (Eyde and Irvin, 1979; Sheppard, 1996) |
| Beatty, NV, USA | n/a | n/a | n/a | n/a | XRD | (Sheppard, 1996) |
| Beaver Rim, WY, USA | n/a | acicular crystals | lacustrine | clin | XRD | (Sheppard, 1996) |
| Beech Creek, USA | Ca | silky fibrous layers on levyne | diagenetic alteration by cool meteoric H_2_O | lev | EMPA, TGA, XRD, OM, SEM | (Bennett and Grose, 1978; Dogan and Dogan, 2008; Passaglia et al., 1998; Sheppard, 1996; Sheppard et al., 1974) |
| Belkin Gravel Pit, ND, USA | n/a | fibrous | n/a | n/a | n/a | (Van Gosen et al., 2013) |
| Benton County, WA, USA | n/a | n/a | n/a | n/a | n/a | (Van Gosen et al., 2013) |
| Boron, CA, USA | n/a | n/a | lacustrine | n/a | XRD | (Sheppard, 1996) |
| Browns Creek, ID, USA | n/a | threadlike fibres or clusters of threadlike fibres | diagenesis in lacustrine deposits; shallow burial depth; low to moderate salinity and alkalinity | clin | XRD, SEM | (Sheppard, 1996, 1991) |
| Buckhorn, NM, USA | n/a | bundles and acicular needle like individual crystals | diagenesis in closed system within a saline alkaline lake | clin, chab, ana | XRD, SEM | (Gude and Sheppard, 1988; Sheppard, 1996) |
| Bullinger Gravel Pit, ND, USA | n/a | fibrous | n/a | n/a | n/a | (Van Gosen et al., 2013) |
| Burke Gravel Pit, ND, USA | n/a | fibrous | n/a | n/a | n/a | (Van Gosen et al., 2013) |
| Buzalski Gravel Pit, ND, USA | n/a | fibrous | n/a | n/a | n/a | (Van Gosen et al., 2013) |
| Cady Mountains, CA, USA | Na | acicular crystal bundles | lacustrine | n/a | XRD, SEM | (Dogan, 2003; Sheppard, 1996) |
| Cape Lookout, OR, USA | n/a | Soft white hairlike crystals or radiating bundles | n/a | clin, mord, phil, dach | EMPA, SEM | (Wise and Tschernich, 1976) |
| Chalk Buttes, MT, USA | n/a | fibrous | n/a | n/a | n/a | (Goodman and Pierson, 2010) |
| Chimney Reservoir, NV, USA | n/a | n/a | lacustrine | n/a | XRD | (Sheppard, 1996) |
| China Lake, CA, USA | n/a | n/a | n/a | phil, ana, clin | Chemical Analysis, XRD | (Hay, 1964; Sheppard, 1996) |
| Clifton, Greenlee County, AZ, USA | n/a | bundles and sprays | n/a | mord, clin, phil | EMPA, XRD, SEM | (Wise and Tschernich, 1976) |
| Davidson Gravel Pit, ND, USA | n/a | fibrous | n/a | n/a | n/a | (Van Gosen et al., 2013) |
| Dripping Spring, AZ, USA | n/a | n/a | lacustrine | n/a | XRD, SEM | (Sheppard, 1996) |
| Durkee, OR, USA | K, Na, Ca | woolly fibres/acicular to rod like individual crystals; bundles of hexagonal rods | low T & P; saline alkaline lake | chab, clin, phil, ana, mord | XRD, SEM, TEM | (Dogan and Dogan, 2008; Eakle, 1898; Kogel et al., 2006; Matassa et al., 2015; Sheppard, 1996; Staples and Gard, 1959) |
| East Short Pine Hills, SD, USA | n/a | fibrous | n/a | n/a | n/a | (Goodman and Pierson, 2010) |
| Eastgate, Churchill County, NV, USA | n/a | prismatic to acicular crystals; aggregates of radiating crystals; pseudomorphs of glass shards | diagenetic alteration in saline alkaline lake; shallow burial | clin, chab, phil, mord | XRD, SEM | (Papke, 1972; Sheppard, 1996) |
| Ehlis Gravel Pit, ND, USA | n/a | fibrous | n/a | n/a | n/a | (Van Gosen et al., 2013) |
| Ekalaka Hills, MT, USA | n/a | fibrous | n/a | n/a | n/a | (Goodman and Pierson, 2010) |
| Del Norte County, CA, USA | n/a | striated, barrel-shaped prisms with a pinacoid, dominantly offretite with small erionite core | n/a | offr | n/a | (Van Gosen et al., 2013) |
| Lander County, NV, USA | n/a | individual acicular crystals | n/a | n/a | SEM, XRD | (Sheppard, 1996) |
| Fort LaClede, WY, USA | n/a | n/a | n/a | clin | XRD | (Sheppard, 1996) |
| Fredricks Gravel Pit, ND, USA | n/a | fibrous | n/a | n/a | n/a | (Van Gosen et al., 2013) |
| Gabbs Valley, NV, USA | n/a | n/a | n/a | n/a | XRD | (Sheppard, 1996) |
| Harney Lake, OR, USA | n/a | individual acicular or prismatic crystals, bundles, and radial aggregates; rarely exhibits split ends or well-formed hexagonal prisms. | diagenesis in closed hydrologic system | ana, chab, clin, mord, phil | XRD, SEM | (Sheppard, 1996) |
| Hawks Butt, WY, USA | n/a | clusters of radiating crystals | n/a | n/a | XRD | (Sheppard, 1996) |
| Hector Mine, CA, USA | n/a | n/a | lacustrine | n/a | XRD | (Sheppard, 1996) |
| Yellowstone Valley, MT, USA | n/a | n/a | lacustrine | clin | XRD | (Sheppard, 1996) |
| Hoff Gravel Pit, ND, USA | n/a | fibrous | n/a | n/a | n/a | (Van Gosen et al., 2013) |
| Horseshoe Dam, AZ, USA | n/a | n/a | lacustrine | n/a | XRD | (Sheppard, 1996) |
| Hungry Valley, NV, USA | n/a | individual acicular to fibrous zeolites | lacustrine | n/a | SEM, XRD | (Sheppard, 1996) |
| Jersey Valley, NV, USA | n/a | individual acicular crystals and rare woolly fibres | n/a | n/a | SEM, XRD | (Deffeyes, 1959; Sheppard, 1996) |
| Joseph School District, OR, USA | n/a | bundles of parallel fibres | n/a | n/a | n/a | (Van Gosen et al., 2013) |
| Pinal County, AZ, USA | n/a | n/a | n/a | n/a | XRD | (Sheppard, 1996) |
| Killdeer Mountain, ND, USA | n/a | single fibres, bundles of fibres and radiating bundles of fibres | diagenesis by alkaline groundwater; lacustrine | clin, chab, offr | SEM-EDS, XRD, EMPA, TEM, PCM | (Goodman and Pierson, 2010; Saini-Eidukat and Triplett, 2014) |
| Kirkland Junction, AZ USA | n/a | n/a | lacustrine | clin | XRD | (Eyde and Irvin, 1979; Sheppard, 1996) |
| Kudrna Gravel Pit, ND, USA | n/a | fibrous | n/a | n/a | n/a | (Van Gosen et al., 2013) |
| Kulish Gravel Pit, AZ, USA | n/a | fibrous | n/a | n/a | n/a | (Van Gosen et al., 2013) |
| Little Badlands, ND, USA | n/a | acicular crystals | n/a | n/a | XRD | (Sheppard, 1996) |
| Long Pines, MT, USA | n/a | fibrous | n/a | n/a | n/a | (Goodman and Pierson, 2010) |
| Malpais Hill, AZ, USA | n/a | acicular, and as radiating clusters | n/a | offr, heu, phil | EMPA, SEM, XRD | (Wise and Tschernich, 1976) |
| Marion County, OR, USA | n/a | columnar bundles of erionite crystals | n/a | n/a | n/a | (Van Gosen et al., 2013) |
| Mauna Kea, HI, USA | n/a | hexagonal rods | n/s | n/a | OM, XRD, Mossbauer spectroscopy, Magnetic Analysis, EMPA, TEM, SEM | (Golden et al., 1993) |
| McDermitt Caldera, OR, USA | n/a | n/a | n/a | n/a | XRD | (Sheppard, 1996) |
| Milwaukie, OR, USA | n/a | needles clustered in the form of a tapering hexagonal prism | n/a | stil, chab, offr, lev | EMPA, XRD, SEM, Optical | (Wise and Tschernich, 1976) |
| Moonstone, WY, USA | n/a | acicular individual crystals and clusters of rod-like and acicular crystals | lacustrine | clin | XRD | (Sheppard, 1996) |
| Mud Hills, CA, USA | n/a | individual acicular and prismatic crystals and rare bundles of radiating crystals | diagenesis in closed lacustrine system; saline alkaline | ana, clin, chab, phil, mord, | XRD, SEM | (Sheppard, 1996; Sheppard and Gude, 1969) |
| Mule Canyon, CA, USA | n/a | n/a | lacustrine | n/a | XRD | (Sheppard, 1996) |
| Newberry Volcano, OR, USA | n/a | acicular bundles of erionite crystals | hydrothermal alteration | ana, chab, fauj | n/a | (Bargar and Keith, 1984) |
| Owens Lake, CA, USA | n/a | n/a | saline alkaline lake | phil, clin, ana | XRD | (Hay, 1964; Sheppard, 1996) |
| Pine Valley, NV, USA | n/a | bundles and aggregates of radiating prismatic crystal | lacustrine | clin, phil | SEM, XRD | (Deffeyes, 1959; Sheppard, 1996) |
| Price Gravel Pit, ND, USA | n/a | fibrous | n/a | n/a | n/a | (Van Gosen et al., 2013) |
| Red Mountain, AZ, USA | n/a | n/a | hydrothermal system in porphyry copper complex | n/a | n/a | (Van Gosen et al., 2013) |
| Redrock Canyon, NV, USA | n/a | n/a | n/a | clin | n/a | (Papke, 1972) |
| Reese River, NV, USA | K | individual acicular crystals and rare woolly fibres. | diagenetic alteration in saline alkaline lake; shallow burial | clin, phil, mord, ana | SEM, XRD | (Deffeyes, 1959; Dogan and Dogan, 2008; Gude and Sheppard, 1981; Papke, 1972; Sheppard, 1996) |
| Reva Gap, SD, USA | n/a | fibrous | n/a | n/a | n/a | (Goodman and Pierson, 2010) |
| Rio Grande River, CO, USA | n/a | individual acicular crystals | n/a | n/a | XRD, SEM | (Sheppard, 1996) |
| Road Mountain, OR, USA | n/a | n/a | n/a | n/a | XRD | (Sheppard, 1996) |
| Rock Island Dam, WA, USA | n/a | fine acicular crystals, isolated and in radiating clusters; white-brown tufted masses of very fine fibres | n/a | paul, offr, phil, clin, chab | EMPA, XRD | (Kamb and Oke, 1960; Tschernich and Wise, 1982; Wise and Tschernich, 1976) |
| Rome, OR, USA | K | individual acicular or rod like crystals and clusters of acicular crystals. | saline alkaline lake | mord, clin | XRD, SEM-EDS, OM, ICP-MS, TEM-EDX, TEM-SAED, Fe Mossbauer spectroscopy | (Ballirano et al., 2009; Dogan et al., 2006; Dogan and Dogan, 2008; Eberly, 1964; Matassa et al., 2015; Pacella et al., 2018; Sheppard, 1996; Sheppard and Gude, 1969) |
| Ryegrass Creek, OR, USA | n/a | n/a | lacustrine | n/a | XRD | (Sheppard, 1996) |
| Sadowky Gravel Pit, ND, USA | n/a | fibrous | n/a | n/a | n/a | (Van Gosen et al., 2013) |
| San Augustin, NM, USA | n/a | acicular to rod like | n/a | clin, ana | XRD, SEM | (Sheppard, 1996) |
| Sheep Mountain, SD, USA | n/a | fibrous and acicular crystals and clusters of radiating fibres | n/a | clin | XRD, SEM | (Sheppard, 1996) |
| Shoshone, CA, USA | n/a | individual acicular and rod-like crystals bundle of acicular crystals and clusters of radiating crystals | n/a | n/a | XRD, SEM | (Sheppard, 1996) |
| Slate Creek, ID, USA | n/a | tiny needles | n/a | n/a | n/a | (Reed, 1937) |
| Slim Buttes, SD, USA | n/a | n/a | n/a | n/a | n/a | (Goodman and Pierson, 2010) |
| S. Desatoya Mountains, NV, USA | n/a | fibrous and radiating crystals | diagenesis at <2500 m depth in open hydrologic system | mord, chab | XRD, OM | (Barrows, 1980; Sheppard, 1996) |
| Spring Creek, NV, USA | n/a | n/a | lacustrine | n/a | XRD | (Sheppard, 1996) |
| Carbon County, UT, USA | n/a | stubby bundles of acicular crystals | n/a | n/a | XRD, SEM | (Sheppard, 1996) |
| Susie Creek, NV, USA | n/a | n/a | n/a | n/a | XRD | (Sheppard, 1996) |
| Thumb Butte, AZ, USA | n/a | radiating sprays or bundles composed of hexagonal erionite prisms | n/a | mord, clin, phil | EMPA, SEM | (Wise and Tschernich, 1976) |
| Tillamook County, OR, USA | n/a | n/a | n/a | n/a | n/a | (Van Gosen et al., 2013) |
| Trinity Range, NV, USA | n/a | n/a | n/a | n/a | XRD | (Sheppard, 1996) |
| Wikieup, AZ, USA | n/a | prismatic or acicular crystals. rare spherulites of erionite | n/a | n/a | XRD | (Sheppard, 1996) |
| Windy Basin, NV, USA | n/a | n/a | lacustrine | n/a | XRD | (Sheppard, 1996) |
| Wrights Point, OR, USA | n/a | n/a | n/a | n/a | XRD | (Sheppard, 1996) |
| Yaquina Head, OR, USA | K | hexagonal erionite needles, spheres of erionite | n/a | clin | EMPA, SEM | (Dogan and Dogan, 2008; Wise and Tschernich, 1976) |
| Yellowstone National Park, WY, USA | n/a | radiating bundles of white fibrous to rod like crystals. | hydrothermal alteration in hot springs | n/a | XRD, SEM | (Bargar and Beeson, 1981; Honda and Muffler, 1970; Sheppard, 1996) |
| Yucca Mountain, NV, USA | n/a | extremely fine-grained with a hairlike morphology | hydrothermal alteration | n/a | SEM, XRD | (Sheppard, 1996) |
